# Evaluation of the Performance of AmpliSeq and SureSelect Exome Sequencing Libraries for Ion Proton

**DOI:** 10.3389/fgene.2019.00856

**Published:** 2019-09-25

**Authors:** Piyush Gampawar, Yasaman Saba, Ulrike Werner, Reinhold Schmidt, Bertram Müller-Myhsok, Helena Schmidt

**Affiliations:** ^1^Research Unit-Genetic Epidemiology, Gottfried Schatz Research Centre for Cell Signaling, Metabolism and Aging, Molecular Biology and Biochemistry, Medical University Graz, Graz, Austria; ^2^Department of Neurology, Clinical Division of Neurogeriatrics, Medical University Graz, Graz, Austria; ^3^Max Planck Institute of Psychiatry, Munich, Germany; ^4^Munich Cluster for Systems Neurology (SyNergy), Munich, Germany; ^5^Institute of Translational Medicine, University of Liverpool, Liverpool, United Kingdom

**Keywords:** exome sequencing, ion proton sequencer, AmpliSeq, SureSelect, library preparation, validation

## Abstract

Library preparation for whole-exome sequencing is a critical step serving the enrichment of the regions of interest. For Ion Proton, there are only two exome library preparation methods available, AmpliSeq and SureSelect. Although of major interest, a comparison of the two methods is *hitherto* missing in the literature. Here, we systematically evaluate the performance of AmpliSeq and SureSelect and present an improved variant calling pipeline. We used 12 in-house DNA samples with genome-wide and exome microarray data and a commercially available reference DNA (NA12878) for evaluation. Both methods had a high concordance (>97%) with microarray genotypes and, when validating against NA12878, a sensitivity and positive predictive values of >93% and >80%, respectively. Application of our variant calling pipeline decreased the number of false positive variants dramatically by 90% and resulted in positive predictive value of 97%. This improvement is highly relevant in research as well as clinical setting.

## Introduction

Genome-wide association studies (GWAS) using microarray-based genotypes in large-scale epidemiological studies played an essential role in the dissection of complex traits (Kiezun et al., 2012). With the evolution of next-generation sequencing (NGS), base-by-base characterization of the human genome became possible. The use of whole-genome sequencing is yet limited due to its high cost. Although whole-exome sequencing (WES) targets less than 2% of the genome (van Dijk et al., 2014), it is a cost-effective way to detect both common and rare variants in protein-coding regions.

There are several NGS platforms available, but the largest share is of Illumina platforms followed by Ion Proton (Goodwin et al., 2016). The major steps in the NGS workflow are library preparation, sequencing, and data analysis. Library preparation is a critical step prior to enrichment, as it includes the targeted probe-based capture or amplification of target regions from genomic DNA. Most of the library preparation methods are designed for Illumina platforms, and several articles compared the performances of these methods (Clark et al., 2011; Lelieveld et al., 2015; Shigemizu et al., 2015). On the contrary, for Ion Proton, only two library preparation methods exist. These are AmpliSeq and SureSelect. Studies comparing these two WES library preparation methods are so far missing.

Previous studies have compared AmpliSeq on Ion platforms with various kits available for Illumina platforms for WES (Loman et al., 2012; Boland et al., 2013; Samorodnitsky et al., 2015). They found that AmpliSeq on Ion platforms is a faster method with high throughput but faces problem in complex genomic regions. A recent in-detail evaluation of AmpliSeq WES using NA12878 reference calls (Damiati et al., 2016) highlighted the limitations of PCR-based target enrichment, provided the list of missed target regions and a filtering strategy to reduce the number of false positives (FPs).

**Figure 1:**
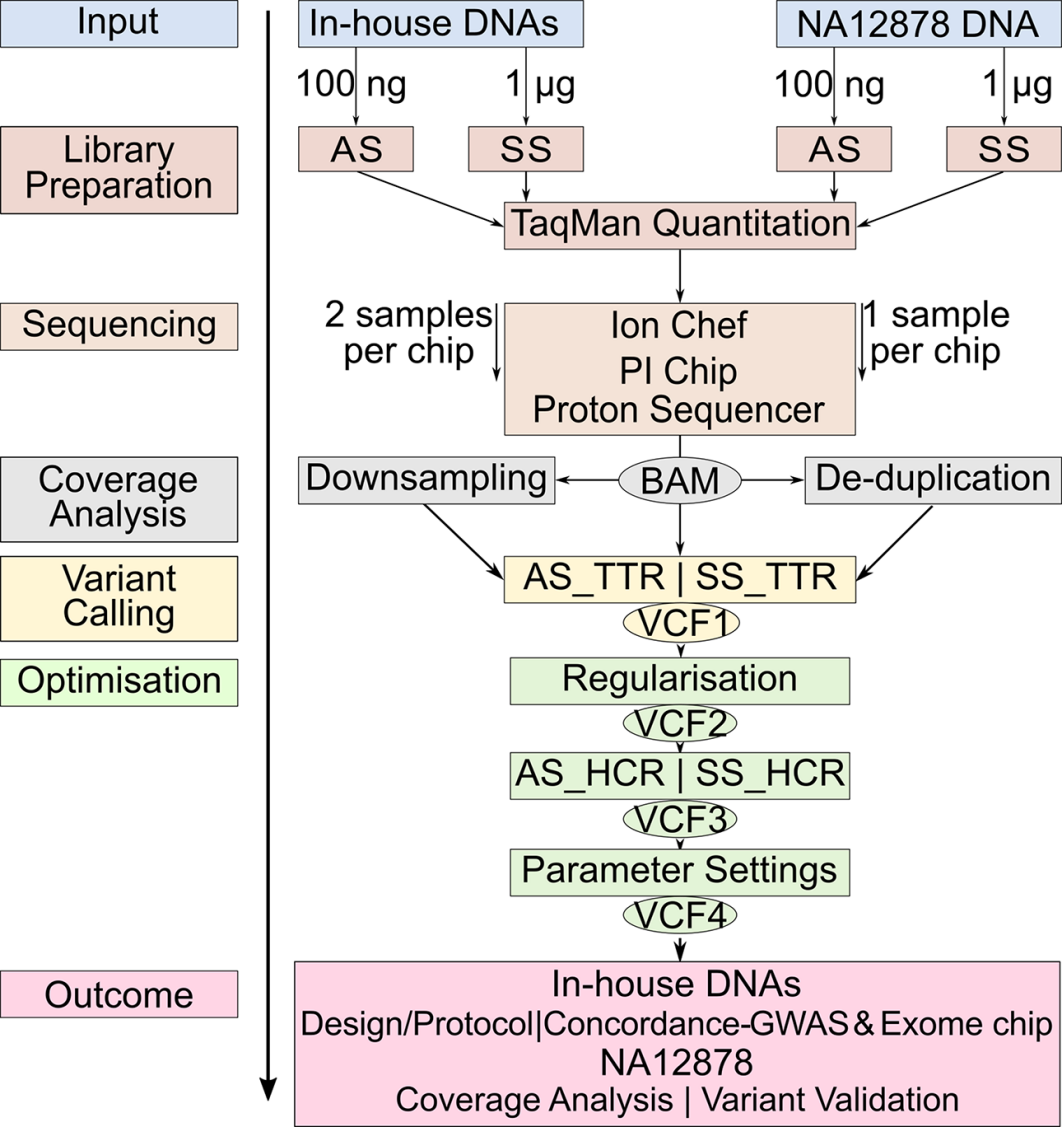
Workflow of the study design. The same color represents the steps at the same level. Identical steps are used to analyze both methods. AS, AmpliSeq; SS, SureSelect; TTR, Total Target Region; ETR, Effective Target Region; OTR, Overlapping Target Region; TPs,True Positives; FNs, False Negatives; FPs, False Positives; PPV, Positive Predictive Value.

Studies comparing the only two library preparation methods, namely, AmpliSeq and SureSelect for Ion Proton, are, however, so far missing. The aims of our study were 1) to compare the performance of AmpliSeq and SureSelect and 2) to develop an optimized protocol for variant calling for WES on Ion Proton platform. We calculated concordance rates between variants detected by sequencing and genome-wide and exome chip genotyping data from 12 in-house DNA samples and validated our sequencing protocol against the well-characterized NA12878 reference DNA v3.3.2 by documenting sensitivity and positive predictive value (PPV) at each optimization step (Zook et al., 2014; Zook et al., 2016) (**Figure 1**).

To our knowledge, this is the first study systematically comparing the performances of AmpliSeq and SureSelect on Ion Proton. We also extend previous findings on the validity of Ion Proton sequencing using AmpliSeq by evaluating different target regions, coverage ranges (44x to 270x) using wet lab sequencing and by manually inspecting all false negative (FN) and FP variants on chromosome (Chr) 1,7,16,19, and X and categorizing them based on their possible causes.

Importantly, AmpliSeq and SureSelect are library preparation and enrichment protocols, which might be chosen prior to sequencing technologies (Illumina vs. Ion Proton). The nature of the sequencing step is, therefore, the object of interrogation. In our study, we aimed to address Ion Proton users and provide a useful point of reference for those who wish to pursue exome sequencing on Ion Proton platform.

## Materials and Methods

### DNA

We used 12 in-house DNA samples previously genotyped by Affymetrix Genome-Wide Human SNP Array 6.0 (n = 11) or Human610-Quad BeadChip (n = 1) (Thermo Fisher Scientific, USA) and by Exome chip Illumina Infinium Exome-24 v1.1 BeadChip array (n = 12) (Illumina Inc., USA). We used the raw data from microarray without any filtration such as minor allele frequency. Altogether six samples (female: 1, males: 5, mean age: 55.7) were withdrawn from the Austrian Stroke Prevention Study (Schmidt et al., 1994), a longitudinal community-dwelling cohort study on brain aging in the city of Graz, Austria. The remaining six samples (females: 2, males: 4, mean age: 74.3) were part of the Prospective Registry on Dementia in Austria (Seiler et al., 2012) and represented patients clinically diagnosed with probable Alzheimer’s dementia. Reference DNA NA12878 (Reference Material 8398) (Zook et al., 2014) was obtained from the “National Institute of Standards and Technology.” In-house DNA samples were extracted from whole peripheral ethylenediaminetetraacetic acid blood using the phenol–chloroform method and stored at -80°C. All DNAs were checked on 1.5% agarose gel and quantified using NanoDrop 3300 fluorospectrometer (Thermo Fisher Scientific, USA) before sequencing.

### Library Preparation and Sequencing

#### Ion AmpliSeq Exome

Library preparation was done with the Ion AmpliSeq^™^ library kit plus (Life Technologies, USA) using 100 ng of DNA (100 ng/µl) according to the manufacturer’s protocol. Briefly, after amplifying the target region using 12 pools of Ion AmpliSeq™ primers (24,000 primer pairs totaling to 294,000), we partially digested the primer sequences and ligated adapters and barcodes to the amplicons. Using the AMPure XP reagent purification system (Beckman Coulter Life Sciences, USA), 50 µl of the purified unamplified library was retrieved. We used Ion AmpliSeq™ Exome RDY plate to amplify eight different and barcoded genomic DNAs at the same time.

#### SureSelect All Human Exome V6

We prepared the library using the SureSelect Target Enrichment System (Agilent Technologies, USA) following the manufacturer’s protocol. After fragmenting 1µg of genomic DNA (100 ng/µl) using Ion Shear Plus Reagents for enzymatic fragmentation, we purified and size-selected the library using AMPure XP beads (Beckman Coulter Life Sciences, USA). We ligated Ion Xpress barcodes and P1 adapters to the end of DNA fragments and then amplified the library. Next, the amplified DNA fragments were hybridized to biotinylated RNA library baits and captured using streptavidin-coated magnetic beads. Finally, captured library fragments were amplified and quality assessed on 2100 Bioanalyser (Agilent Technologies, USA).

We used Ion library TaqMan™ quantitation kit (Life Technologies, USA) on the 7900 real-time PCR system (Applied Biosystems, USA) for quantitation of both unamplified libraries. We did template preparation using Ion PI™ Hi-Q™ chemistry (Life Technologies, USA). We loaded 50 pM of each library on Ion Chef ™ Instrument (Life Technologies, USA) for template enrichment. We performed quality control to assess templating efficiency of Ion spheres using Qubit™ 2.0 Fluorometer (Thermo Fischer Scientific, USA). We loaded prepared libraries onto Proton PI chips v3 (two samples/chip for in-house DNAs; one sample/chip for reference DNA) and sequenced them on Ion Proton using PI™ Hi-Q™ sequencing 200 chemistry (Life Technologies, USA) aiming for read length of 260 bp and 520 flow cycles.

### Data Analysis

We used Ion Torrent Suite^1^ version 5.4 (Life Technologies, USA) to analyze the data. We used Torrent Mapping Alignment Program version 5.2 for alignment against human hg19 assembly and Torrent Variant Caller (TVC) version 5.4 under the default low stringency settings to call variants. We analyzed the variants in the library-specific total target region (TTR). Also, for AmpliSeq, we used the effective target region (ETR) (**Supplementary Datasheet 2**), which is introduced by the manufacturer in the default Ion Torrent pipeline to exclude poor performing regions enriched for FPs or having low coverage. We downloaded the RefSeq-, Ensembl-, and University of California—Santa Cruz (UCSC)-defined coding regions from UCSC genome browser in the form of BED files (20/04/2017).

We used v3.3.2 of high-confidence calls vcf file of NA12878 from Genome in the Bottle project (Zook et al., 2014) downloaded from their ftp server for validation of our data. For optimizing our pipeline, we used the high-confidence region (HCR), provided as BED file (Zook et al., 2014; Zook et al., 2016). HCR specifies those regions in the genome where genotypes can be called confidently. These regions were generated after arbitrating between 11 whole-genome and 3 exome data sets from 5 sequencing platforms and 7 mappers by Zook et al. (2014) and carefully filtering uncertain sites. These regions were provided as BED file. We intersected the target regions from AmpliSeq and SureSelect with the provided HCR to get the HCRs in the respective target design.

We used bedtools (Quinlan and Hall, 2012) to manipulate BED files and vcf files and bcftools^2^ to calculate the true positives (TPs), FNs, and FPs. We used vcflib^3^ vcfallelicprimitives module to generate phased genotypes and vt to regularize the variants (Tan et al., 2015). The vcfallelicprimitive module splits the multiple representations of a single record in a vcf file into multiple lines. This is necessary as indels and complex variants are frequently called differently depending upon the aligner used to create BAM files. It results in the representation of multi-nucleotide variants as two SNVs. The vt tool performs normalization by left alignment and presents a variant in as few nucleotides as possible. The normalization helps to compare the variants called by the different variant caller to minimize errors.

For *in silico* downsampling, we used samtools view -s option that selects the desired number of reads from a big BAM file (Li et al., 2009). We used the tools picard^4^, samtools, and a java-based tool “MarkDupbyStartEnd”^5^ to remove duplicates. We applied different combinations of parameters for variant calling on TVC to get a balance between FNs and FPs. Finally, we visualized all FNs and FPs on Chr 1, 7, 16, 19, and X using Integrative Genomics Viewer (IGV) (Robinson et al., 2011). We used Rstudio for statistical computation and graphics^6^.

### Categorization of False Negatives and False Positives

We manually inspected all FNs and FPs on Chr 1, 7, 16, 19, and X. We selected Chr 1, as it is the largest chromosome and has the highest number of FNs, Chr 7 and 16 as they have a high density of exonic monomer repeats, Chr 19 as it has the highest density of sequence repeats, and Chr X as representation of a sex chromosome (Subramanian et al., 2003). We classified FNs due to possible causes related to 1) library-derived issues such as coverage, genotype, and read quality or a combination of these and 2) sequencer-derived issues such as location in a homopolymer region and signal shifts or both. When we cannot identify the reason behind an FN, we categorized it as unknown. We classified FPs into six categories by inspecting each position on IGV. 1) Strand bias: ≤2% of reads of alternate alleles are from one strand, 2) Read end: a variant present within five nucleotides at the end of a read, 3) Low quality: the quality of the variant call was less than 20, 4) Homopolymer: a variant inside or next to a repeat stretch of four or more nucleotides, 5) Mixed allele: more than one alternate allele was present at that particular position, 6) Unknown: variants failed to be categorized under the mentioned five categories.

### Z Score for Coverage Comparison and Evenness of Coverage

We divided read depth into 45 categories. For the lower end of read distribution (0X–10X), we used an increment of 5X, through 10X–400X an increment of 10X, and above 400X that of 200X. This allowed us a high-resolution comparison of the distribution of reads in the low read depth region (<10X) and in the callable region. The callable range is defined between 5X and 400X and was set by the manufacturer in order to reduce the computation time. Next, we calculated the difference in the coverage between the AmpliSeq and SureSelect for each category. We computed the normalized difference in coverage as follows:

Diffcoverage = AmpliSeqcoverage - SureSelectcoverage

Z = (Diffcoverage- Mean(Diffcoverage)/SD(Diffcoverage)

Where SD(Diffcoverage) is the standard deviation of the difference in coverage.

Z > 0 means higher AmpliSeq coverage than SureSelect and vice versa.

We calculated the evenness of coverage for both libraries to compare the target enrichment by dividing the per base coverage by the average depth.

## Results

### Comparison of AmpliSeq and SureSelect Laboratory Protocol and Design

A detailed description of the design and protocol is presented in **Table 1**. Briefly, by design, AmpliSeq TTR targets 57,742,646 bp and SureSelect TTR 60,456,963 bp. The overlapping target region (OTR) is 43,173,762 bp. AmpliSeq TTR covers 91.1, 88.6, and 87.9% while SureSelect TTR 87.9, 87.8, and 87.4%, of RefSeq-, Ensembl-, and UCSC-defined coding regions, respectively. The size of AmpliSeq ETR is 46,347,343 bp and covers 86.3, 83.7, and 83.1% of the RefSeq-, Ensemble-, and UCSC-coding regions. We found that in both the libraries, the missed regions were exclusively located in the coding region. AmpliSeq by design missed 3,016,767, while SureSelect missed 4,227,905 bases from RefSeq-coding regions.

**Table 1:** Comparison of laboratory protocols and design of AmpliSeq and SureSelect library preparation methods.

		AmpliSeq	SureSelect
**Protocol**	Enrichment approach	PCR	Hybridization
	DNA input	100 ng	1 µg
	Steps in library preparation	3	8
	DNA fragmentation	NA	Enzymatic fragmentation
	Target selection	Amplification using primers	Hybridization with RNA library baits
	Incubation time	∼4 h	∼26 h
	Library Preparation time	6 h	2.5 days
**Target Region**	Total target region	57.74 MB	60.45 MB
	RefSeq coding	32.30 MB (91.13%)	31.15 MB (87.88%)
	UCSC coding	32.57 MB (88.65%)	32.26 MB (87.78%)
	Ensembl coding	32.40 MB (87.90%)	32.23 MB (87.44%)
	Effective target region	46.35 MB	NA
	RefSeq coding	30.58 MB (86.28%)	NA
	UCSC coding	30.76 MB (83.72%)	NA
	Ensembl coding	30.62 MB (83.07%)	NA

NA, not applicable; MB, million bases.

### Analysis of In-House DNA Samples

The mean number of reads was 34.2 million for AmpliSeq and 39.8 million for SureSelect. The mean read depth values were 92X for AmpliSeq and 69X for SureSelect. Out of the total number of reads produced by each method, 94% in AmpliSeq and 86% in SureSelect were mapped to their respective target regions, a difference that was statistically significant (p < 0.0001). The percentage of bases covered >50X and >5X were 63 and 97.3% with AmpliSeq and 50.3 and 97.9% with SureSelect (**Supplementary Table 1**). Under the default low-stringency settings of TVC, the mean numbers of variants including singletons over all samples were 51,413 in AmpliSeq and 51,783 in SureSelect TTR (**Supplementary Table 2**). The mean concordance rates were around 98% with exome chip and 99.5% with the GWAS chip genotypes for both libraries (**Supplementary Table 3**). We disregarded homozygous reference calls. Concordance rates were somewhat lower (AmpliSeq: 95%, SureSelect: 92%) for rare variants (MAF < 0.05%).

**Table 2:** Variant validation of default TVC output (VCF1) against NA12878 truth set.

	Total Variants	Truth set	TPs	FNs	FPs	Sensitivity	PPV
AmpliSeq							
Total Variants	54,351	49,340	45,946	3,394	8,405	93.12%	84.54%
Total SNVs	50,913	45,092	43,840	1,252	7,073	97.22%	86.11%
Exonic SNVs	19,650	16,964	16,588	376	3,062	97.78%	84.42%
Total Indels	3,436	4,248	2,106	2,142	1,330	49.58%	61.29%
Exonic indels	539	329	231	98	308	70.21%	42.86%
SureSelect							
Total Variants	54,934	46,982	43,929	3,053	11,005	93.50%	79.97%
Total SNVs	52,013	43,367	42,230	1,137	9,783	97.38%	81.19%
Exonic SNVs	19,171	16,120	15,846	274	3,325	98.30%	82.66%
Total Indels	2,921	3,614	1,699	1,915	1,222	47.01%	58.17%
Exonic indels	312	277	195	82	117	70.40%	62.50%

Truth set—Variants in v3.3.2 of high-confidence calls VCF of NA12878 from Genome in the Bottle project, SNVs,Single Nucleotide Variants; TPs,True Positives; FNs, False Negatives; FPs, False Positives; PPV, Positive Predictive Value; TVC, Torrent Variant Caller, VCF1—This corresponds to **Figure 1**.

### Analysis of NA12878 Reference DNA

#### Coverage

**Figure 2:**
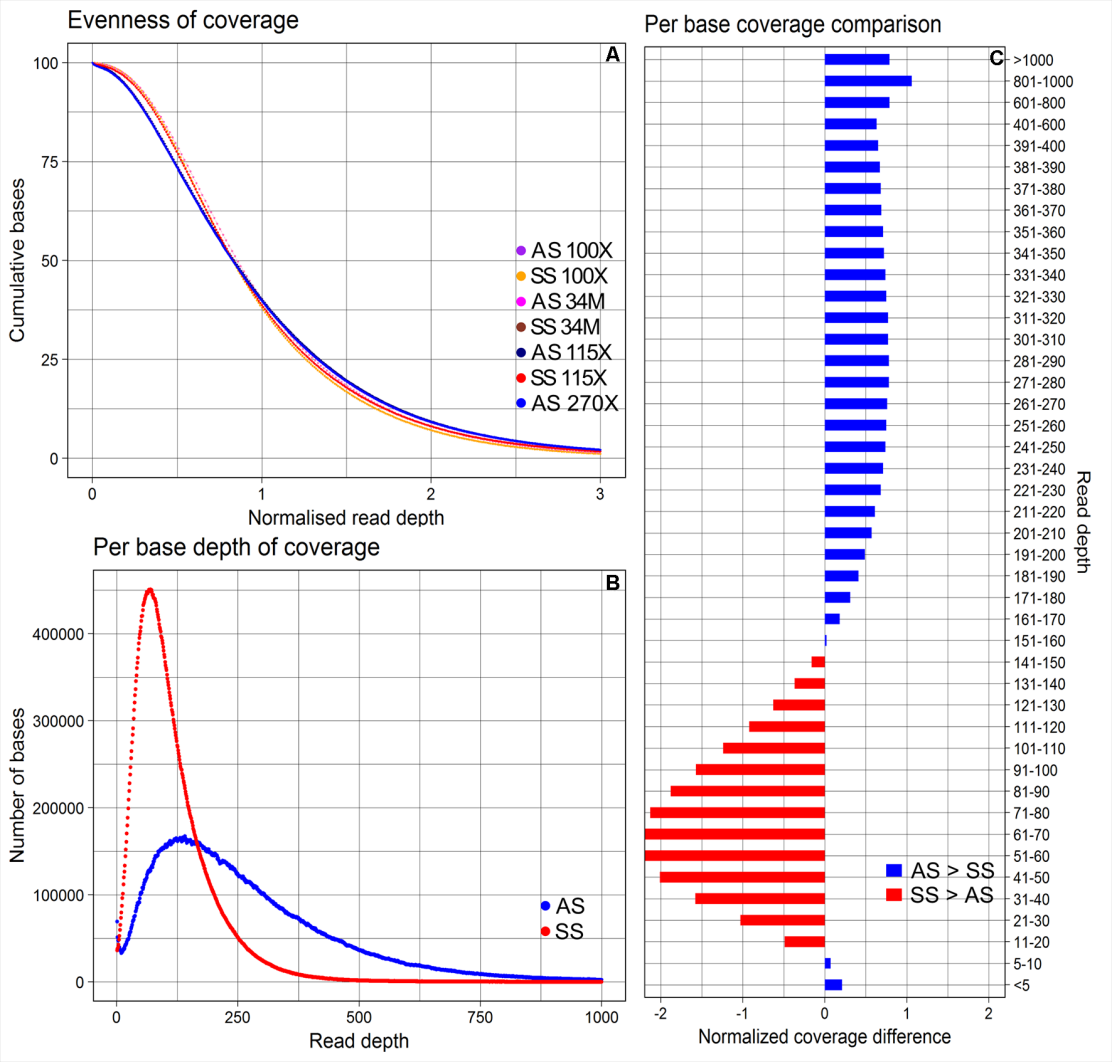
Evenness of coverage, per base depth of coverage and its comparison between AmpliSeq and SureSelect methods. **(A)** Evenness of coverage plotted for original and downsampled BAM files **(B)** Scatter plot showing the distribution of per base coverage of AmpliSeq and SureSelect till 1000X read depth. **(C)** A bar chart is showing the difference in coverage after dividing the depth of coverage into 45 groups and normalization. SureSelect covers more bases in the coverage range of 11X to 150X than AmpliSeq. AS, AmpliSeq; SS, SureSelect.

The average depths of coverage were 270X for AmpliSeq and 115X for SureSelect. The proportions of bases covered between 5X and 400X were 79.4% for AmpliSeq and 98.5% for SureSelect, covered >400X was profoundly larger for AmpliSeq (19.9%) than for SureSelect (0.9%) and covered less than 5X was similarly low for both (<1%) (**Supplementary Table 4**). Both libraries covered approximately 40% of the total targeted bases with more than the average depth, however, in AmpliSeq percentage of bases covered less than 10X was higher than that in SureSelect (**Figure 2**).

#### Variant Detection

By using AmpliSeq, we identified 54,351 variants, while by using SureSelect, the number of detected variants was 54,934 (VCF1). The overall sensitivity and PPV were 93.1 and 84.5% for AmpliSeq and 93.5 and 80% for SureSelect, respectively. The sensitivity and PPV for detecting SNVs were higher than those for detecting indels by using both libraries (**Table 2**). Performing variant calling on 34 million randomly selected reads from both libraries to reduce the coverage bias resulted in 53,068 and 52,918 variants within AmpliSeq and SureSelect TTRs. AmpliSeq had a sensitivity of 91.8% and PPV of 85.3%, whereas SureSelect had a sensitivity of 91% and PPV of 80.8% (**Supplementary Table 5**).

#### Optimization-Variant Calling Pipeline

**Figure 3:**
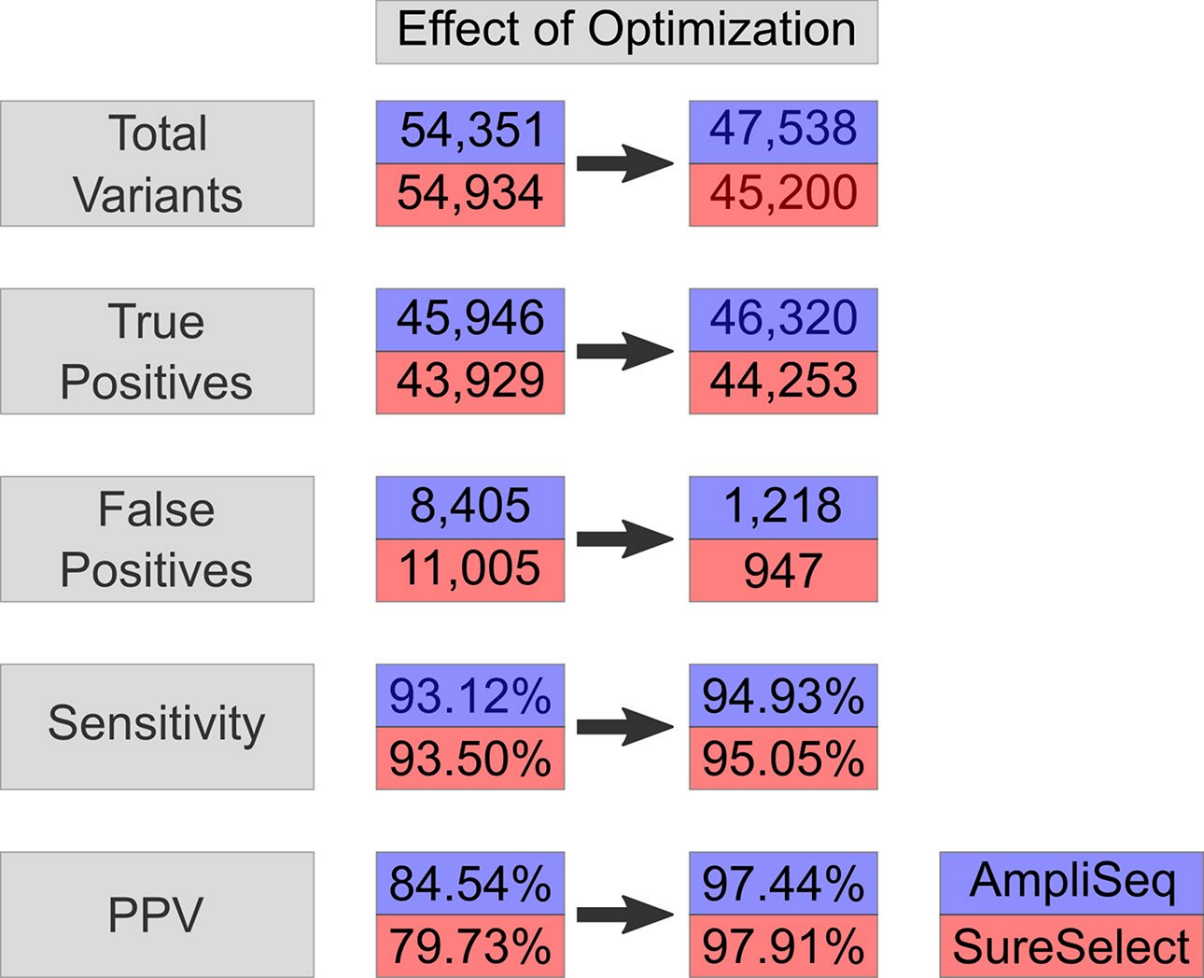
Effect on optimization-variant calling pipeline. Effect of optimization steps shown on total variants, true positives, false positives, sensitivity, and PPV in AmpliSeq and SureSelect. Blue represents AmpliSeq and red SureSelect. PPV, positive predictive value.

Upon regularization, as recommended by Zook et al., 2014, the number of TPs increased by 714 to 46,660 using AmpliSeq and by 622 to 44,551 using SureSelect (VCF2). At the same time, FPs increased by 176 to 8,581 and by 275 to 11,280, respectively. By using the HCR BED file to exclude difficult-to-sequence regions, the number of FPs decreased significantly from 8,581 to 1,218 in AmpliSeq and from 11,005 to 947 in SureSelect, while the number of TPs decreased minimally by 0.7% (VCF3) (**Table 3**). The sensitivity and PPV for detection of SNVs were 98.7 and 98.3% for AmpliSeq and 98.8 and 98.6% for SureSelect, respectively. Corresponding values for indels were 52.7 and 82.7% for AmpliSeq and 49.1 and 84.4% for SureSelect, respectively (**Figure 3**).

**Table 3:** Variant validation in various steps of optimization using NA12878 truth set.

Steps	Total Variants	Truth set	TPs	FNs	FPs	Sensitivity	PPV
AmpliSeq							
TTR (VCF1)	54,351	49,340	45,946	3,394	8,405	93.12%	84.54%
RG (VCF2)	55,241	49,340	46,660	2,680	8,581	94.57%	84.47%
HCR (VCF3)	47,538	48,796	46,320	2,476	1,218	94.93%	97.44%
SureSelect							
TTR (VCF1)	54,934	46,982	43,929	3,053	11,005	93.50%	79.97%
RG (VCF2)	55,831	46,982	44,551	2,431	11,280	94.83%	79.80%
HCR (VCF3)	45,200	46,557	44,253	2,304	947	95.05%	97.91%

Truth set—Variants in v3.3.2 of high-confidence calls VCF of NA12878 from Genome in the Bottle project, TPs, True Positives; FNs, False Negatives; FPs, False Positives; PPV, Positive Predictive Value, VCF1–3—This corresponds to **Figure 1**, TTR, Total Target Region; RG, Regularization; HCR, High-Confidence Region.

The total number of TP, FN, and FP indels were 2,121, 1,904, and 445 by AmpliSeq and 1,710, 1,771, and 335 in SureSelect, respectively. The overlapping positions of TP, FN, and FP indels were 870, 642, and 31 between the two libraries, respectively (**Supplementary Tables 6** and **7**). Out of 1,261 indels missed by AmpliSeq, 132 were detected by SureSelect, while out of 1,128 indels missed by SureSelect, 247 were detected by AmpliSeq (**Supplementary Figure 1**).

Last, we repeated variant calling by changing the default parameter settings in a stepwise manner while keeping HCR as a target region for each library. While SureSelect had the best performance with the default parameter settings, AmpliSeq performed best when the parameter “minimum allele frequency” was changed to 0.2 (step 2). In AmpliSeq, PPV improved from 97.4 to 98.1% with a reduction of sensitivity by 0.3% (**Supplementary Table 8** and **Supplementary Figure 2**). To equalize read depth over two methods, we downsampled both libraries to 34 million reads as well as to an average depth of 100X and observed a similar increase in PPV to 98% and a minimum reduction in sensitivity (**Supplementary Table 5**).

#### AmpliSeq Effective Target Region

In ETR, AmpliSeq detected a total of 38,651 variants with 33,119 being TPs, 1,251 FNs, and 5,532 FPs. Using regularization and restricting the analysis to HCR resulted in a substantial reduction of FPs by 91% and increased PPV to 98.5% while maintaining sensitivity at 98% (**Supplementary Table 9**).

### Variant Detection in RefSeq Coding Region and Overlapping Target Region

In the RefSeq-coding region, we detected 17,836 variants using AmpliSeq and 17,312 using SureSelect out of the expected 19,270 coding variants present in NA12878 truth set. After regularization and using HCR, sensitivity increased slightly from 92.6 to 93.4% for AmpliSeq and from 89.8 to 90.5% for SureSelect, whereas PPV increased considerably from 82.6 to 98.1% and 80 to 98.1%, respectively (**Table 4**).

**Table 4:** Comparing performance of AmpliSeq vs. SureSelect within RefSeq-coding region and overlapping target region.

**RefSeq-coding region**	Steps	Total Variants	Truth set	TPs	FNs	FPs	Sensitivity	PPV
**AmpliSeq**							
TTR (VCF1)	21,584	19,270	17,836	1,434	3,748	92.56%	82.64%
RG (VCF2)	21,878	19,270	18,087	1,183	3,791	93.86%	82.67%
HCR (VCF3)	18,331	19,270	18,009	1,261	322	93.46%	98.24%
**SureSelect**							
TTR (VCF1)	21,649	19,270	17,312	1,958	4,337	89.84%	79.97%
RG (VCF2)	21,943	19,270	17,523	1,747	4,420	90.93%	79.86%
HCR (VCF3)	17,747	19,270	17,443	1,827	304	90.52%	98.29%
**OTR 115X**	**AmpliSeq**							
	TTR (VCF1)	35,093	32,213	30,367	1,846	4,723	94.27%	86.53%
	RG (VCF2)	35,550	32,213	30,788	1,425	4,762	95.58%	86.60%
	HCR (VCF3)	31,161	31,979	30,611	1,368	550	95.72%	98.23%
	**SureSelect**							
	TTR (VCF1)	36,228	32,213	30,651	1,562	5,577	95.15%	84.61%
	RG (VCF2)	36,744	32,213	31,067	1,146	5,677	96.44%	84.55%
	HCR,(VCF3)	31,478	31,979	30,896	1,083	582	96.61%	98.15%

Overlapping target region (OTR) —this is the common region covered by both AS and SS designs. This region is 43.17 Mb, RefSeq-coding region—this is the coding region from RefSeq database, downloaded from UCSC table browser on 20/04/2017. NA12878 truth set has 19,270 variants, Truth set—Variants in v3.3.2 of high-confidence calls VCF of NA12878 from Genome in the Bottle project, SNVs, Single Nucleotide Variants; TPs, True Positives; FNs, False Negatives; FPs, False Positives; PP, Positive Predictive Value, VCF1–3—This corresponds to **Figure 1**, RG, Regularization; HCR, High-Confidence Region.

Next, we compared the performances of both libraries in the OTR of 43.2 million base pairs at the same average depth of 115X. After regularization and restricting analyses to HCR, we saw a similar improvement as that of TTR or RefSeq-coding region. The sensitivity of both methods was around 95%, and PPV was improved from 85 to 98% (**Table 4**). In OTR, out of the total TPs called by each method, 30,266 were shared, leaving 1–2% of variants specific to each library (**Supplementary Figure 3**).

### De-Duplication

Removing duplicates using picard and samtools resulted in an 88% loss of reads in AmpliSeq and 30% loss in SureSelect. Therefore, we did not perform variant calling in AmpliSeq. Using the tool “MarkDupbyStartEnd,” the loss was 13% in AmpliSeq and 0.1% in SureSelect. There was no change in the performance of either library by applying de-duplication strategies, and sensitivity remained around 94% and PPV 98% for both libraries (**Supplementary Table 10**).

### Exploration of False Negatives and False Positives

Manual inspection of all FNs on Chr 1, 7, 16, 19, and X (**Supplementary Figure 4**) showed that the FN SNVs were mainly due to library-derived issues (74–95%) whereas indels were due to all three, namely library derived, sequencer derived or both issues (14–58%) in both libraries (**Supplementary Tables 11**–**17**). We validated our classification of FNs by scrutinizing shared FN positions between the two methods (Chr 1: 35 and Chr X: 18) and found that, except for two positions, the classification was concordant. Among the clearly classifiable FP SNVs (**Supplementary Figure 5**), strand bias was the major cause in AmpliSeq (51–61%), while in SureSelect, homopolymers played a prominent role (18-50). Homopolymer-related issues explained most FP indels (44–79%) in both methods (**Supplementary Tables 18**–**24**). We did not find any major differences between the causes of FNs or FPs in the respective TTRs or library-specific regions in either library.

### Effect of Increasing Average Read Depth on AmpliSeq Performance

Increasing average depth from 44X to 270X had a significant effect on the per base coverage (**Supplementary Figure 6**). Importantly, raising average read depth led to decrease in bases covered <5X (3,188,163 to 400,116) but, on the same time also, to a disproportional increase in the number of bases covered >400X (19,602 to 11,494,834). This resulted in a relevant decrease in the number of bases in the callable range (5X–400X from 55,687,667 at 44X to 45,880,764 at 270X). Sensitivity increased significantly from 86.2 to 94.9%, while the change in PPV was negligible (96.9 to 97.4%) (**Supplementary Table 25**).

## Discussion

In the present study, we compared the performance of the AmpliSeq and the SureSelect library preparation methods, the two presently available methods for WES on the Ion Proton platform. By design, AmpliSeq covers a slightly larger proportion of the RefSeq-, Ensembl-, and UCSC-coding regions than does SureSelect. When comparing with exome chip genotype data, both show excellent concordance rates of 97%. Validating the two methods against NA12878 truth set revealed a comparable sensitivity (93%) but a higher PPV for AmpliSeq (84.5%) than for SureSelect (80%). By applying regularization using HCR and altering the default parameter settings for variant calling, we were able to reduce the number of FPs by approximately 90% and reach a final sensitivity of 95% and PPV of 97% for both methods.

### Protocol and Design

Owing to its PCR-based design, the laboratory protocol of the AmpliSeq method is considerably faster (6 h), consists of fewer preparation steps, and requires less hands-on time than SureSelect. Starting from genomic DNA, it allows the identification of exonic variants within 48 h. The low amount of input material required is a further advantage over SureSelect. Therefore, when the amount of starting material is low and time is a constraint, AmpliSeq is the method of choice.

### Microarray Concordance

Both methods have similarly excellent concordance rates of >97% against exome chip and >99% against GWAS chip genotype data. The comparable concordance rates despite the lower average read depth in SureSelect is probably due to a more favorable distribution of the per-base coverage. As we describe in the NA12878 sequencing results, the proportion of bases in the callable range (5-400x) was significantly higher in SureSelect (98.49%) than in AmpliSeq (79.49%). The callable range was set up by the manufacturer to reduce the computation time. Moreover, in case of very rare variants (MAF < 0.05%) sequencing detected on average 90% more variants than the exome chip.

### Variant Detection

Validating sequencing data against microarrays has been shown to overestimate sensitivity and is prone to errors at indels and phased genotypes (Zook et al., 2014). Therefore, as recommended (Zook et al., 2014), we also used high-confidence genotypes from the NA12878 truth set to validate the two libraries and found that sensitivity was comparable for both methods (93.1 vs. 93.5%) while PPV was relevantly higher for AmpliSeq (84.5 vs. 80%).

#### Optimization-Variant Calling Pipeline

In order to improve the validity of variant calling, we used 1) regularization, 2) restriction to HCR, and 3) changing default parameter settings. The regularization had the strongest effect on the reduction of FNs by 20% in both libraries. This was due to breaking down complex variants to their simplest form, generating phased genotypes. The most remarkable reduction in FPs (AmpliSeq by 85.8%, SureSelect by 91.6%) could be achieved by restricting the analyses to the HCR. This effect is due to the exclusion of complex, difficult to sequence areas such as simple repeats, tandem duplications, regions inside structural variants, etc. of the genome (Zook et al., 2014). Recently, Samorodnitsky et al. (2015) also reported that PCR-based methods are more prone to errors in complex genomic regions. However, using HCR reduces the target coding region by 5.5 MB in AmpliSeq and 6.8 MB in SureSelect with important implications for the identification of pathogenic variants in rare diseases. We, therefore, provide a list of regions excluded while using HCR (**Supplementary file**). If pathogenic variants are expected in these regions, we recommend variant calling against the TTR using a list of all known pathogenic variants (hotspot list). This strategy delivers genotypes at all positions given on the hotspot list even when the individual is homozygous for the reference allele.

Fine-tuning by changing parameter settings for variant calling reduced FPs significantly by 27% only in AmpliSeq but had practically no effect in SureSelect (**Supplementary Table 26**). This difference is probably due to the fact that in SureSelect, strand bias and minimal allele frequency, which are influenced by PCR, are of less concern.

We found that for the detection of SNVs, both AmpliSeq and SureSelect have high sensitivity and PPV; however, for detecting indels, sensitivity and PPV are significantly reduced for both methods. Our estimates for the performance of the AmpliSeq method are in line with the previous study (Damiati et al., 2016); however, for comparing our results on SureSelect performance on Ion Proton, no study is available so far.

#### AmpliSeq Effective Target Region

When using our optimization pipeline with ETR instead of TTR, the number of FPs and FNs was substantially reduced, resulting in a relevant improvement in PPV from 85.7 to 98.5%. A drawback of using ETR was, however, the significant reduction of TPs by 28%. This is an important novel finding, as the use of ETR by default is recommended by the manufacturer. We, however, strongly recommend using our pipeline together with TTR instead of ETR.

#### RefSeq-Coding Region and Overlapping Target Region

Focusing on the RefSeq-coding region and OTR, we could achieve a more valid comparison of two methods. In the RefSeq-coding region, AmpliSeq detected 3% more variants than SureSelect. This was probably due to the fact that AmpliSeq by design covers 3.2% more of the RefSeq-coding region than SureSelect does. In OTR, the performances of both methods were identical.

Importantly, our data show that it is possible to combine the genotypes from both methods for meta-analysis, as we found that within OTR; ∼98% TP variants were shared by both libraries, and the number of library-specific variants were reduced to a minimum.

### De-Duplication

As AmpliSeq is a PCR-based library preparation method, PCR duplicates removal using standard practices resulted in the loss of most reads (88%), whereas, in SureSelect, it did not improve the outcome. Moreover, as suggested by Ebbert et al. (2016), duplicate removal had a minimal effect on downstream variant calling. Therefore, we do not suggest duplicate removal on either of the libraries on Ion Proton.

### Exploration of False Negatives and False Positives

Irrespective of the library used, library-related issues dominated FN SNVs, whereas, for FN indels, library as well as sequencer-related problems contributed evenly. FP SNVs in AmpliSeq were predominantly due to strand bias, a PCR-related issue; hence, increasing the parameter “minimum allele frequency” led to a significant reduction of FPs by 27%. Homopolymers were the major cause of FP indels in both methods. Therefore, regardless of the chemistry used for library preparation, FN and FP indels on Ion Proton were mainly due to the sequencing technology. This is in line with previous findings showing a high rate of errors in complex genomic regions using Torrent technology (Samorodnitsky et al., 2015). FPs categorized as “unknown” were located mainly in complex regions.

Many of these FN and FP variants could be overcome by the use of third-generation sequencing, as these techniques lack amplification steps and therefore hinders introduction of chimeric reads, variation in repeat size, and under-representation of GC-rich/poor regions (Ardui et al., 2018), hence, allow an accurate detection of variants in difficult-to-sequence regions. Especially, as reported by Huddleston et al. (2017), by using third-generation sequencing, a significant improvement in detecting indels >7 bp and structural variants <1 kilobase, which account for the majority of differences between genomes, is expected.

### Effect of Coverage on AmpliSeq Performance

Contrary to our expectations, increasing the mean read depth from 44X to 270X did not result in a reduction of FNs and FPs. Rather, we observed that the performance was reaching a plateau between 80-90X. Indeed, an increase in average read depth exponentially increased the number of bases covered >400X but not those covered <5X (**Supplementary Table 25**). This is most probably due to PCR-based biases that occur during the target selection and library enrichment. These results echoed the previous finding using *in silico* downsampling (Damiati et al., 2016). Those regions covered by AmpliSeq with less than 5X, SureSelect design targeted 62%. Out of these 62% targeted regions, 72% were covered by ≥5X by SureSelect (**Supplementary Figure 7**).

### Mapping and Variant Calling Pipelines

As most of the Proton users apply the user-friendly Torrent Mapping Alignment Program + TVC pipeline provided by the manufacturer, we aimed to evaluate the two library preparation methods using this pipeline. Torrent Suite is optimized for Ion Torrent data and is constantly updated, resolving the weaknesses associated in each version. We did not test alternative variant calling pipelines because previous reports clearly showed that for the analysis of data from Ion Torrent platforms, Torrent Suite is the most sensitive and specific (Caboche et al., 2014; Vanni et al., 2015; De Summa et al., 2017).

### Strengths and Weaknesses

The strength of our study lies in the stepwise and thorough comparison of the AmpliSeq and SureSelect methods, including their design, protocols, sequencing performances, and variant calling. We performed 24 sequencing experiments on in-house DNAs with GWAS and exome chip genotype data, and six sequencing runs on the reference NA12878 DNA. We estimated the validity of the methods by calculating the concordance rates against microarray genotypes as well as the number of TPs, FNs, FPs, sensitivity, and PPV using high-confidence genotypes from NA12878. We optimized the variant calling pipeline by using regularization, HCR, and alternative parameter settings. This finally led to a substantial improvement in sensitivity and PPV for both methods. We manually inspected all FNs and FPs on Chr 1, 7, 16, 19, and X and categorized them based on their most likely origin. We explored the potential of increasing average read depth using AmpliSeq as a measure to improve variant calling and provide evidence that an average read depth of 80–100X, which is achievable by loading two samples/PI chip, represents an optimal setting.

We did not compare AmpliSeq and SureSelect with WES library preparation methods available for the Illumina platform. Instead, we especially addressed the scientist using Ion Proton. Due to limited resources, we were only able to explore the effect of coverage in AmpliSeq but not in SureSelect. We also did not explore the regions failed to be sequenced by either library. It was, however, already done for AmpliSeq by a recent study (Damiati et al., 2016). We did not aim to compare the two library preparation methods for CNV analysis at present. The reason for our decision is the lack of available tools that allow a direct comparison of the two methods for CNV detection. Recently, SureSelect Human All Exon V7 became available, which covers 96% of RefSeq-coding region and could be used on Ion Proton. We did not explore the performance of this library preparation kit in our protocol. Importantly, our pipeline was optimized for hg19 reference assembly. Uplifting hg19 data to GRCh38 led to a significant reduction of the target regions for both libraries. AmpliSeq HCR decreased from 53.6 to 49.7 MB and SureSelect from 54.2 to 50.19 MB. Moreover, we found that the variants in the truth set in these target regions also decreased from 48,796 to 45,749 in AmpliSeq HCR and from 46,557 to 43,453 in SureSelect HCR. This is probably due to the fact that both design and protocols are optimized for hg19. In case GRCh38 data are used, a re-optimization of our protocol is required.

This is the first study evaluating the performance of AmpliSeq and the SureSelect, the only two library preparation methods available for WES on Ion Proton and is therefore of major interest to the users of this technology. Both methods reach high validity, but AmpliSeq is faster, requires significantly less input material, and outperforms SureSelect in the number of variants detected in the RefSeq-coding region. By applying our newly developed variant calling pipeline, a further significant increase in sensitivity to 95% and PPV to 98% and a dramatic reduction in the number of FPs by 90% can be achieved. This improvement is highly relevant both in research as well as clinical settings.

## Data Availability

The datasets generated and analyzed are available in the Sequence Read Archive repository (https://www.ncbi.nlm.nih.gov/sra/SRP155134).

## Ethics Statement

The studies involving human participants were reviewed and approved by the ethics committee of the Medical University of Graz. The patients/participants provided their written informed consent to participate in this study.

## Author Contributions

PG—Sequenced and analyzed the data and wrote the paper. YS—Analyzed the data. UW—Collected data and critically read the manuscript. BM-M and RS—Critically read the manuscript. HS—Study design, obtained the funds, and wrote the paper.

## Funding

The research reported in this article was funded by the Austrian Science Fund grant number P13180, and P20545-B05, by the Austrian National Bank Anniversary Fund, P15435, and the Austrian Ministry of Science under the aegis of the EU Joint Programme—Neurodegenerative Disease Research—www.jpnd.eu. The project is supported through the following funding organizations under the aegis of EU Joint Programme—Neurodegenerative Disease Research—www.jpnd.eu: Australia, National Health and Medical Research Council, Austria, Federal Ministry of Science, Research and Economy; Canada, Canadian Institutes of Health Research; France, French National Research Agency; Germany, Federal Ministry of Education and Research; Netherlands, The Netherlands Organisation for Health Research and Development; United Kingdom, Medical Research Council. This project has received funding from the European Union’s Horizon 2020 research and innovation program under grant agreement no. 643417. Part of this study was funded by the grant Franz Lanyar Stiftung from the Medical University of Graz, Austria and by the Austrian Atherosclerosis Society. PhD position for PG is supported through the PhD program “Molecular Medicine” of Medical University of Graz, Graz, Austria.

## Conflict of Interest Statement

The authors declare that the research was conducted in the absence of any commercial or financial relationships that could be construed as a potential conflict of interest.

## Footnotes

1 Ion Torrent Suite Software 5.4 https://assets.thermofisher.com/TFS-Assets/LSG/manuals/MAN0016409_TorrentSuite5_4Help pdf.

2 Samtools/bcftools. *GitHub*. https://github.com/samtools/bcftools

3 vcflib/vcflib. GitHub. https://github.com/vcflib/vcflib

4 Picard http://broadinstitute.github.io/picard

5 MarkDupsByStartEnd Github. https://github.com/dariober/Java-cafe/tree/master/MarkDupsByStartEnd

6 RStudio Team. RStudio: Integrated Development for R. RStudio, Inc., Boston, MA http://www.rstudio.com/.
