# Supplementary material for: Evaluation of the Performance of AmpliSeq and SureSelect Exome Sequencing Libraries for Ion Proton
Source: Front Genet. 2019 Sep 25;10:856. doi: 10.3389/fgene.2019.00856 (PMC6774276; doi:10.3389/fgene.2019.00856)
Supplement: Supplementary file 1 [file DataSheet_1.pdf]

Supplementary Material

1. Supplementary Figures and Tables

1.1 Supplementary Figures

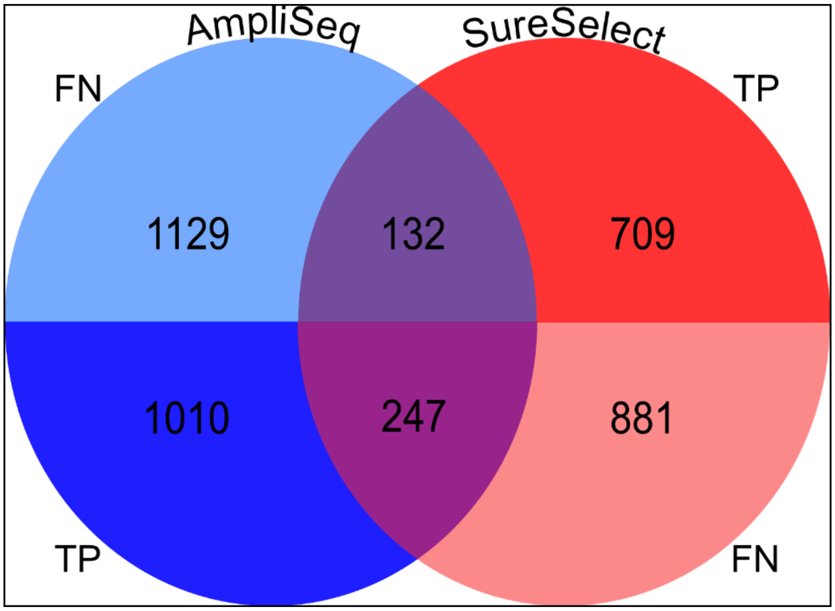

Supplementary Figure 1: Comparison of true positive and false negative indels. Blue represents TP indels only identified by AmpliSeq, violet represents TP indels in AmpliSeq which were missed by SureSelect, and sky blue represents FN indels in AmpliSeq. Red represents TP indels only detected by SS, purple TP indels in SureSelect which were missed by AmpliSeq and Light red represents SureSelect FN indels. TP: true positives, FN: false negatives

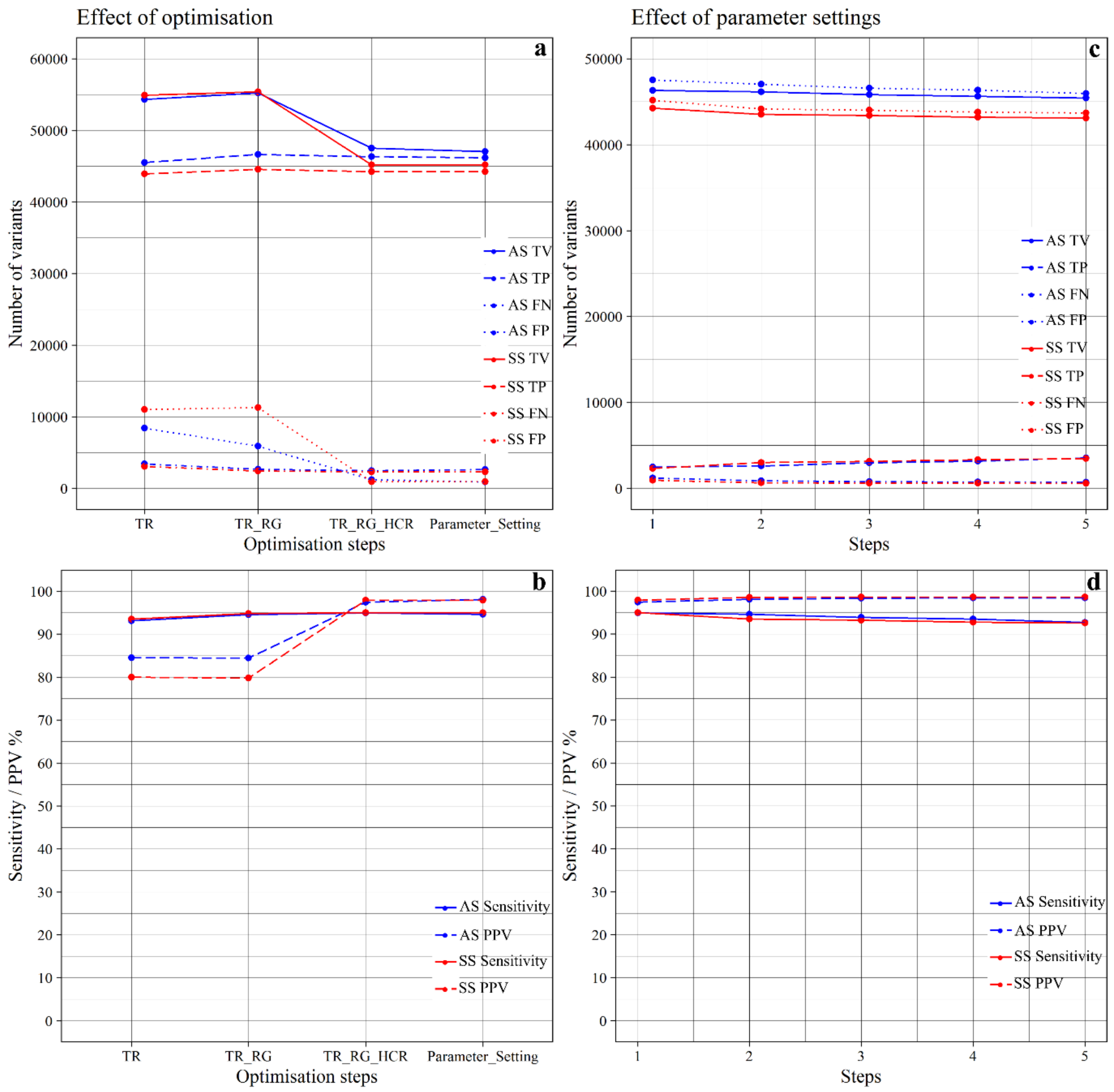

Supplementary Figure 2: Effect of stepwise optimization strategies on AmpliSeq (blue) and SureSelect (red) performance. Effect of 3 step optimization on a) total variants (TV), true positives (TP), false positives (FP) and false negatives (FN) b) on sensitivity and positive predictive value (PPV). Effect of low to high stringency 5 step parameter setting on the variants is shown in c and d.

AS-AmpliSeq SS-SureSelect

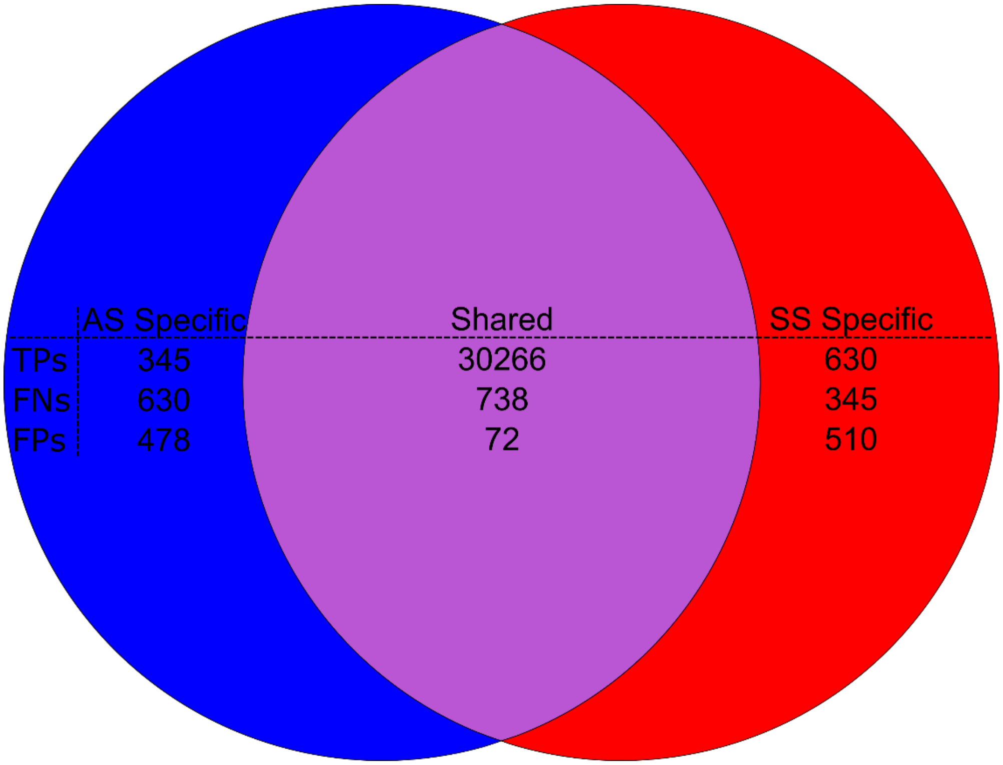

Supplementary Figure 3: Number of variants shared between two libraries and specific to each library in the overlapping target region. In the overlapping target region of 43.17 million bases, both libraries shared 98% of variants detected. We performed this analysis on after equalizing depth of coverage to 115X for both libraries.

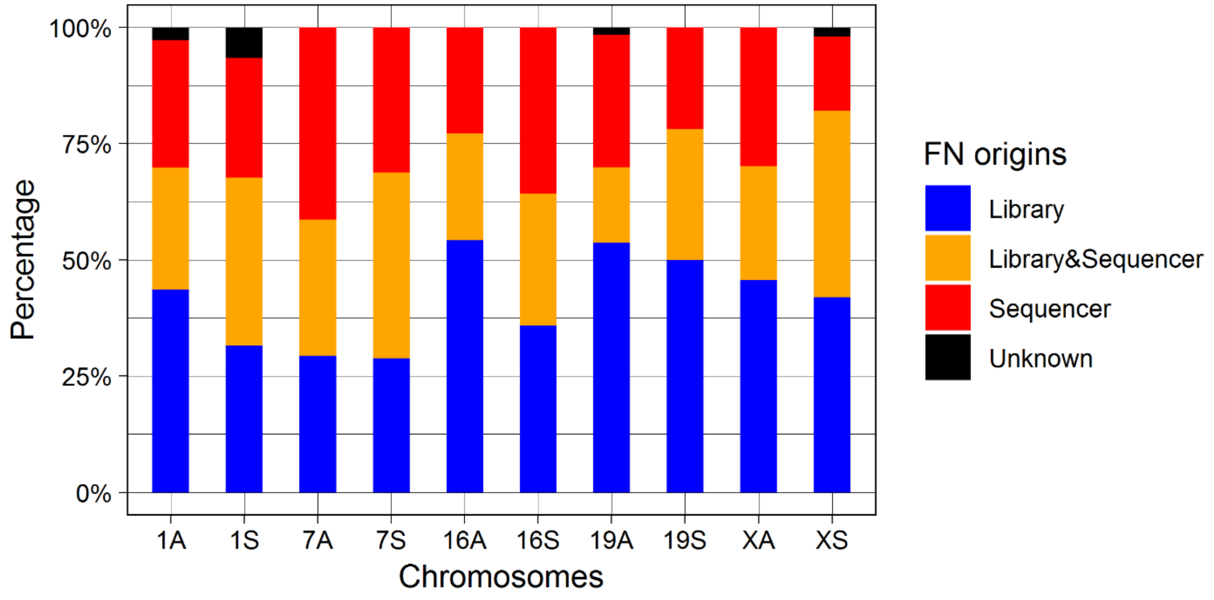

Supplementary Figure 4: Comparison of the percentage of false negatives in a library derived, sequencer derived, both, and unknown categories. Each bar represent a chromosome and A or S represent AmpliSeq or SureSelect. On each bar, 4 colors represent the percentage of false negatives in 4 categories.

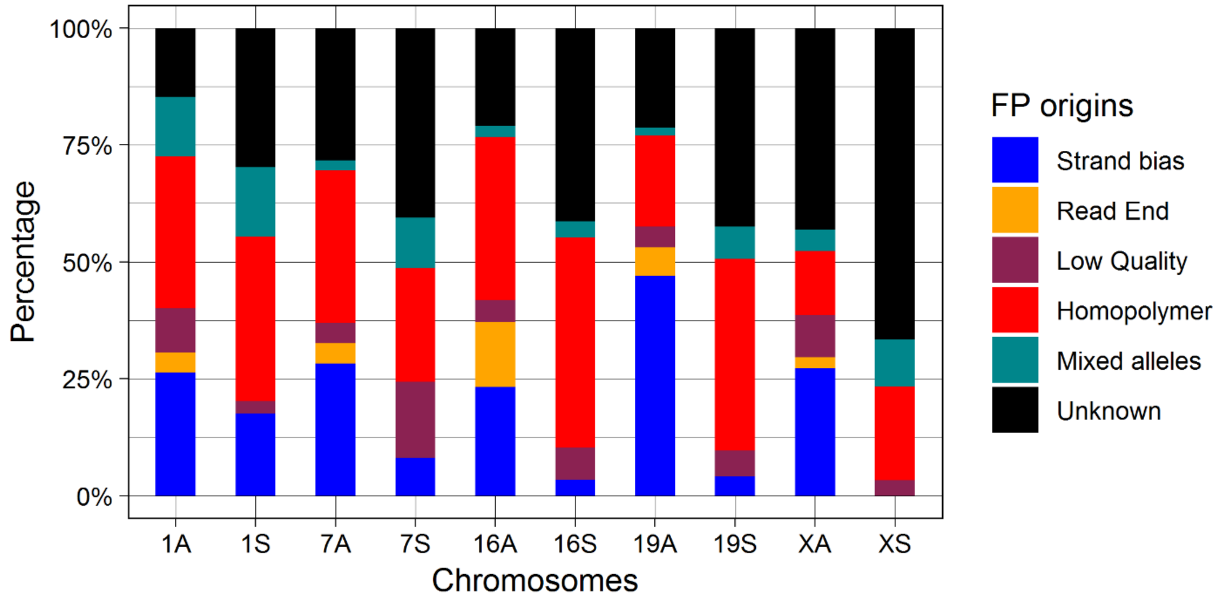

Supplementary Figure 5: Comparison of the percentage of false positives in strand bias, read end, low quality, homopolymer, mixed alleles and unknown categories. Each bar represent a chromosome and A or S represent AmpliSeq or SureSelect. On each bar, 6 colors represent the percentage of false negatives in 6 categories.

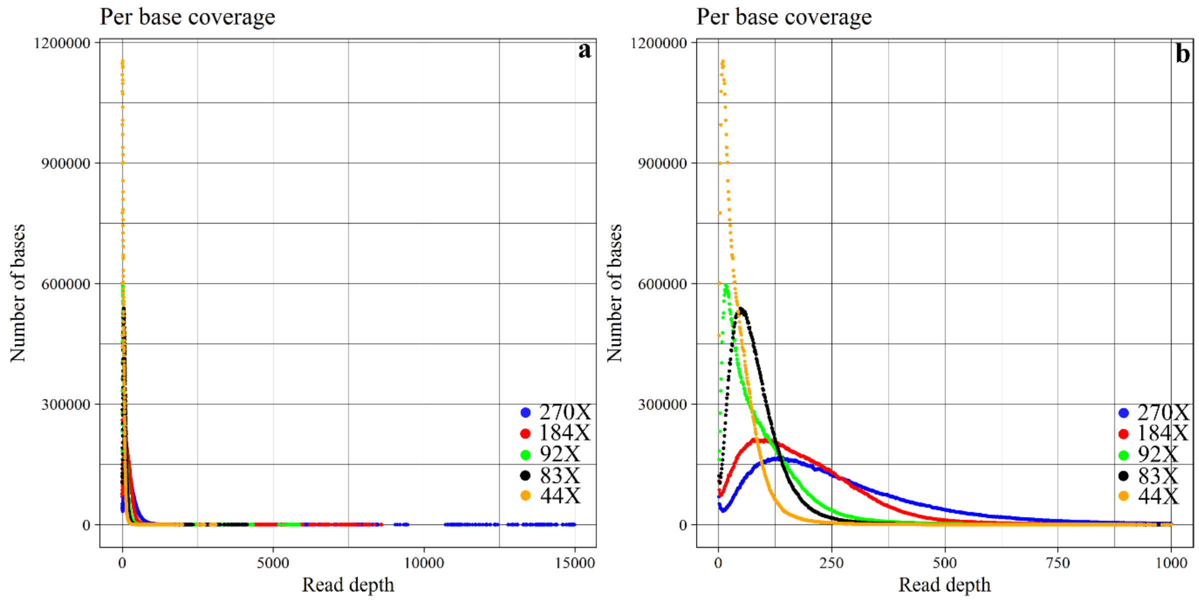

Supplementary Figure 6: Comparison between AmpliSeq runs at a different average depth of coverage. (a)Scatter plot showing the distribution of per base coverage of 5 sequencing run of AS over total read depth. The average depth of coverage of 270x (blue), 184X (red), 92X (green), 83X (black) and 44X (yellow) are seen. (b) Scatter plot showing the distribution of per base coverage of AmpliSeq and SureSelect till 1000X read depth. With the increase in average depth of coverage more than 100X, the proportion of a relative number of bases covered with more than 400X increases more than between 5X to 400X, leading to increase in average depth of coverage.

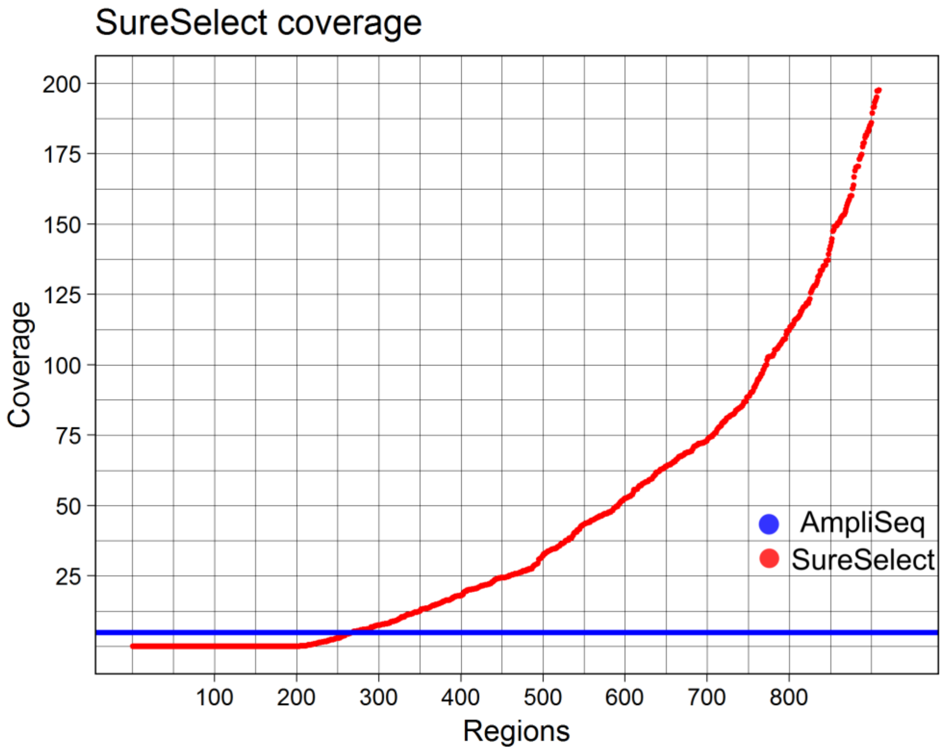

Supplementary Figure 7: Coverage of SureSelect on low covered AmpliSeq regions. SureSelect design covered 122,709 bases (spanned over 935 regions) out of 196,820 bases which are covered with < 5X by AmpliSeq. Out of these 935 regions in SureSelect, 268 regions had coverage of < 5X and remaining 667 regions of ≥5X.

1.2 Supplementary Tables

Supplementary Table 1: Target enrichment efficiency with AmpliSeq and SureSelect methods

Sample ID	GWAS SNPs passing QC	Exomechip SNPs passing QC	AmpliSeq						SureSelect					
			Total Reads	Mapped Reads	Mapped Reads %	Reads on Target %	Mean Read Length	Mean Read Depth	Total Reads	Mapped reads	Mapped Reads %	Reads on Target %	Mean Read Length	Mean Read Depth
26500	501,288	242,688	36,844,784	36,514,871	99.1	94.9	173	102	33,403,340	33,267,894	99.6	87.0	115	51
59301	501,288	242,688	29,945,578	29,789,626	99.5	95.3	177	84	41,990,192	41,802,769	99.6	84.9	149	75
90901	501,288	242,688	34,829,164	34,582,922	99.3	95.1	176	98	44,110,131	43,935,592	99.6	84.8	151	80
152301	501,288	242,688	35,414,963	34,943,911	98.7	93.6	157	87	40,376,249	40,200,406	99.6	93.6	149	87
168002	501,288	242,688	20,064,165	19,832,304	98.8	89.1	180	56	53,862,426	53,563,459	99.4	85.8	149	98
801019	491,791	244,688	35,704,021	35,500,942	99.4	95.8	180	104	37,144,327	36,983,305	99.6	87.2	102	51
801020	491,791	NA	50,119,270	49,782,561	99.3	95.9	181	146	40,164,626	39,991,973	99.6	85.8	110	58
801024	491,791	244,688	28,924,948	28,632,133	99.0	95.1	152	69	33,479,566	33,339,769	99.6	82.4	147	58
801042	491,791	244,688	30,488,385	30,178,632	99.0	95.7	153	74	35,129,719	34,927,962	99.4	82.9	145	60
801052	491,791	244,688	27,983,305	27,560,123	98.5	93.4	139	60	45,745,431	45,491,089	99.4	84.3	146	80
801058	491,791	NA	41,756,741	41,288,706	98.9	90.2	184	118	26,070,226	25,979,250	99.7	84.5	150	47
13	501,288	NA	38,495,116	38,051,197	98.8	94.9	171	105	46,329,776	46,148,050	99.6	86.8	138	82
Mean± SD			34,214,203± 7,617,405	33,889,119± 7,577,988	99±0.3	94.1±2.2	169±15	92±26	39,817,167± 7,371,996	39,635,960± 7,324,890	99.6±0.1	85.8±2.9	133±18	69±17

p value < 0.0001

p value - 0.001

p value - 0.002

p value - <0.0001

p value - <0.0001

p value- 0.67

NA: not available

Supplementary Table 2: Variants detected with AmpliSeq and SureSelect libraries using different target regions

Sample ID	AmpliSeq TTR	SureSelect TTR	AmpliSeq ETR	Difference AmpliSeq TTR vs AmpliSeq ETR (%)
26500	51,704	48,629	37,679	14,025 (27.13)
59301	50,343	53,562	36,576	13,767 (27.35)
90901	52,026	53,492	37,523	14,503 (27.88)
152301	51,875	52,748	37,316	14,559 (28.07)
168002	48,383	52,668	35,625	12,758 (26.37)
809019	52,097	48,087	37,662	14,435 (27.72)
809020	52,325	49,622	37,722	14,603 (27.91)
801024	51,279	52,422	37,198	14,081 (27.46)
801042	51,053	52,416	37,081	13,972 (27.37)
801052	51,018	54,284	37,105	13,913 (27.27)
801058	52,996	51,895	38,186	14,810 (77.95)
13	51,860	51,566	37,547	14,313 (27.60)
Mean±SD	51,413±1,180	51,783±1,982	37,268±658	14,145±542 (27.50±0.47)

TTR –Total Target Region
ETR –Effective Target Region

Supplementary Table 3: Concordance between sequencing and microarray genotyping in detail

Exome chip (n=9)						
Sample ID	AmpliSeq			SureSelect		
	Common Positions	Concordant variants	Concordance rate	Common Positions	Concordant variants	Concordance rate
26500	7,142	7,009	98.1%	7,245	7,062	97.5%
59301	6,851	6,709	97.9%	7,329	7,154	97.6%
90901	7,093	6,966	98.2%	7,406	7,229	97.6%
152301	6,950	6,799	97.8%	7,302	7,113	97.4%
168002	6,791	6,669	98.2%	7,377	7,226	98.0%
801019	7,113	6,965	97.9%	7,174	6,974	97.2%
801020	NA					
801024	7,080	6,946	98.1%	7,402	7,236	97.8%
801042	6,995	6,858	98.0%	7,389	7,219	97.7%
801052	7,021	6,836	97.4%	7,465	7,277	97.5%
801058	NA					
13	NA					
Mean ± SD	7,004±121	6,862±102	97.97±0.26%	7,343±91	7,166±99	97.58±0.22%
**GWAS chip (n=12)**						
Sample ID	AmpliSeq			SureSelect		
	Common Positions	Concordant variants	Concordance rate	Common Positions	Concordant variants	Concordance rate
26500	3,759	3,740	99.5%	4,826	4,790	99.3%
59301	3,523	3,494	99.2%	4,924	4,892	99.4%
90901	3,779	3,768	99.7%	5,034	5,018	99.7%
152301	3,698	3,681	99.5%	5,055	5,024	99.4%
168002	3,541	3,526	99.6%	4,946	4,926	99.6%
801019	3,626	3,612	99.6%	4,657	4,629	99.4%
801020	3,711	3,700	99.7%	4,787	4,767	99.6%
801024	3,628	3,604	99.3%	4,891	4,861	99.4%
801042	3,697	3,673	99.4%	4,991	4,966	99.5%
801052	3,626	3,596	99.2%	4,976	4,958	99.6%
801058	3,718	3,715	99.9%	4,918	4,879	99.2%
13	6,518	6,474	99.3%	8,300	8,257	99.5%
Mean ± SD	3,664±83	3,646±87	99.51±0.23%	4,910±116	4,883±118	99.45±0.16%

*Data from two exome chip samples (801058 & 801020) was not used due to its low quality and sample 13 didn't have exome chip data.
*Genotyped with Affymetrix Genome-Wide Human SNP Array 6.0 Illumina Infinium Exome-24 v1.1 BeadChip and Illumina Human610-Quad BeadChip
*The concordance rate was calculated using VCF 1 (figure 1)

Supplementary Table 4: Coverage per base level in AmpliSeq and SureSelect libraries with NA12878 DNA

Bases covered	AmpliSeq	%	SureSelect	%
<5X	400,116	0.69	371,807	0.61
<10X	587,610	1.02	671,090	1.11
5X-10X	220,562	0.38	378,922	0.63
5X-400X	45,847,696	79.40	59,541,459	98.49
10X-400X	45,660,202	79.08	59,242,176	97.99
>400X	11,494,834	19.91	543,697	0.90
Total target bases	57,742,646		60,456,963	
Average read depth	270X		115X	

Supplementary Table 5: Variant validation against NA12878 truth set in downsampled BAM

	Steps	Total Variants	Truthset	TPs	FNs	FPs	Sensitivity	PPV
**34 Million reads**	**AmpliSeq**							
	TTR (VCF1)	53,068	49,340	45,267	4,073	7,801	91.75%	85.30%
	RG (VCF2)	53,890	49,340	45,949	3,391	7,941	93.13%	85.26%
	HCR (VCF3)	46,697	48,796	45,613	3,183	1,084	93.48%	97.68%
	**SureSelect**							
	TTR (VCF1)	52,918	46,982	42,763	4,219	10,155	91.02%	80.81%
	RG (VCF2)	53,705	46,982	43,346	3,636	10,359	92.26%	80.71%
	HCR (VCF3)	43,951	46,557	43,057	3,500	894	92.48%	97.97%
**100X Average depth**	**AmpliSeq**							
	TTR (VCF1)	53,121	49,340	45,298	4,042	7,823	91.81%	85.27%
	RG (VCF2)	53,941	49,340	45,981	3,359	7,960	93.19%	85.24%
	HCR (VCF3)	46,745	48,796	45,648	3,148	1,097	93.55%	97.65%
	**SureSelect**							
	TTR (VCF1)	54,612	46,982	43,734	3,240	10,878	93.09%	80.08%
	RG (VCF2)	55,511	46,982	44,353	2,629	11,158	94.40%	79.90%
	HCR (VCF3)	45,043	46,557	44,054	2,503	989	94.62%	97.80%

Truth set-Variants in v3.3.2 of high confidence calls VCF of NA12878 from Genome in the Bottle project, SNVs- Single Nucleotide Variants, TPs- True Positives, FNs- False Negatives, FPs- False Positives, PPV-Positive Predictive Value, VCF1-3 - This corresponds to figure 1, TTR - Total Target Region, RG – Regularization, HCR - High Confidence Region

Supplementary Table 6: Variant validation of optimized output (VCF3) against NA12878 truth set

	Total Variants	Truthset	TPs	FNs	FPs	Sensitivity	PPV
**AmpliSeq**							
Total Variants	47,535	48,796	46,320	2,476	1,215	94.93%	97.44%
Total SNVs	44,969	44,771	44,199	572	770	98.72%	98.29%
Exonic SNVs	16,953	16,895	16,737	158	216	99.06%	98.73%
Total Indels	2,566	4,025	2,121	1,904	445	52.70%	82.66%
Exonic indels	326	309	230	79	96	74.43%	70.55%
**SureSelect**							
Total Variants	45,194	46,556	44,253	2,303	941	95.05%	97.92%
Total SNVs	43,169	43,075	42,543	532	626	98.76%	98.55%
Exonic SNVs	16,187	16,054	15,966	88	221	99.45%	98.63%
Total Indels	2,025	3,481	1,710	1,771	315	49.12%	84.44%
Exonic indels	255	263	188	75	67	71.48%	73.73%

Truth set-Variants in v3.3.2 of high confidence calls VCF of NA12878 from Genome in the Bottle project, SNVs- Single Nucleotide Variants, TPs- True Positives, FNs- False Negatives, FPs- False Positives, PPV-Positive Predictive Value

Supplementary Table 7: Indel detection by AmpliSeq and SureSelect

Indels	AmpliSeq specific	SureSelect specific	Overlapping indels
True Positives	1,251	841	870
False Negatives	1,261	1,128	642
False Positives	437	310	31

Supplementary Table 8: Effect of parameter settings on variant detection

Step	Total Variants	TPs	FNs	FPs	Sensitivity	PPV
**AmpliSeq**						
1	47,538	46,320	2,476	1,218	94.93%	97.44%
2	47,061	46,177	2,619	884	94.63%	98.12%
3	46,594	45,832	2,964	762	93.93%	98.36%
4	46,374	45,639	3,157	735	93.53%	98.42%
5	45,957	45,248	3,548	709	92.73%	98.46%
**SureSelect**						
Step	Total Variants	TPs	FNs	FPs	Sensitivity	PPV
1	45,191	44,250	2,307	941	95.05%	97.92%
2	44,173	43,542	3,015	631	93.52%	98.57%
3	44,027	43,422	3,135	605	93.27%	98.63%
4	43,806	43,209	3,348	597	92.81%	98.64%
5	43,690	43,103	3,454	587	92.58%	98.66%

Step 1 - MiAF=0.1 MiCo=5 MiCo/str=0 MxStrBi=0.98
Step 2 - MiAF=0.2 MiCo=5 MiCo/str=0 MxStrBi=0.98
Step 3 - MiAF=0.2 MiCo=5 MiCo/str=2 MxStrBi=0.8
Step 4 - MiAF=0.2 MiCo=10 MiCo/str=3 MxStrBi=0.8
Step 5 - MiAF=0.2 MiCo=10 MiCo/str=5 MxStrBi=0.8
MiAF- minimum allele frequency
MiCo-minimum coverage
MiCo/str- minimum coverage on either strand
MxStrBi- maximum strand bias
TPs- True Positives
FNs- False Negatives
FPs- False Positives
PPV-Positive Predictive Value

Supplementary Table 9: Variant validation against NA12878 truthset in AmpliSeq ETR

**AmpliSeq**							
Steps	Total Variants	Truthset	TPs	FNs	FPs	Sensitivity	PPV
ETR (VCF1)	38,651	34,370	33,119	1,251	5,532	96.36%	85.69%
RG (VCF2)	39,112	34,370	33,559	811	5,553	97.64%	85.80%
HCR (VCF3)	33,888	34,118	33,383	735	505	97.85%	98.51%

Truthset- Variants in v3.3.2 of high confidence calls vcf file of NA12878 from Genome in the Bottle project
TPs- True Positives
FNs- False Negatives
FPs- False Positives
PPV-Positive Predictive Value

Supplementary Table 10: Variant validation against NA12878 after duplicate removal.

	Total Variants	Truthset	TPs	FNs	FPs	Sensitivity	PPV
**AmpliSeq**							
PICARD	88% loss of reads						
Samtools	87.8% loss of reads						
Start End	46,720	48,796	45,609	3,187	1,111	93.47%	97.62%
**SureSelect**							
PICARD	44,662	46,557	43,886	2,691	796	94.26%	98.26%
Samtools	44,662	46,557	43,886	2,691	796	94.26%	98.26%
Start End	45,041	46,557	44,053	2,504	988	94.62%	97.81%

Truthset- Variants in v3.3.2 of high confidence calls vcf file of NA12878 from Genome in the Bottle project
TPs- True Positives
FNs- False Negatives
FPs- False Positives
PPV-Positive Predictive Value

Supplementary Table 11 : Distribution of false negatives from chromosome 1 in various categories

**AmpliSeq**																
	Library derived							Sequencing derived						Both	Unknown	Total
	Min coverage	Min coverage on either strand	Max strand bias	Min quality	Min relative read quality	Mixed	Total	Maximum homopolymer length	Excess outlier reads	Maximum common signal shift	Maximum reference / variant signal shift	Mixed	Total			
SNVs	2(5%) (67%)	1(2%) (20%)	2(5%) (40%)	2(5%) (29%)	0 (0%) (0%)	34(83%) (60%)	41 (53%)	0 (0%) (0%)	0 (0%) (0%)	9(90%) (32%)	0 (0%) (0%)	1(10%) (9%)	10 (20%)	1 (2%)	3 (60%)	55 (31%)
Indels	1(3%) (33%)	4(11%) (80%)	3(8%) (60%)	5(13%) (71%)	1(3%) (100%)	23(62%) (40%)	37 (47%)	8(21%) (100%)	2(5%) (100%)	19(49%) (68%)	0 (0%) (0%)	10(25%) (91%)	39 (80%)	46 (98%)	2 (40%)	124 (69%)
Total	3(4%)	5(6%)	5(6%)	7(9%)	1(1%)	57(73%)	78	8(16%)	2(4%)	28(57%)	0(0%)	11(22%)	49	47	5	179
Group Total	78							49						47	5	179
Group %	43.58							27.37						26.26	2.79	
**SureSelect**																
	Library derived							Sequencing derived						Both	Unknown	Total
	Min coverage	Min coverage on either strand	Max strand bias	Min quality	Min relative read quality	Mixed	Total	Maximum homopolymer length	Excess outlier reads	Maximum common signal shift	Maximum reference / variant signal shift	Mixed	Total			
SNVs	0 (0%) (0%)	0 (0%) (0%)	6(21%) (86%)	1(4%) (14%)	0 (0%) (0%)	21(75%) (81%)	28 (65%)	0 (0%) (0%)	0 (0%) (0%)	0 (0%) (0%)	0 (0%) (0%)	0 (0%) (0%)	0 (0%)	0 (0%)	2 (22%)	30 (22%)
Indels	0 (0%) (0%)	3(20%) (100)	1(7%) (14%)	6(40%) (86%)	0 (0%) (0%)	5(33%) (19%)	15 (35%)	10(29%) (100%)	3(9%) (100%)	11(30%) (100%)	1(3%) (100%)	10(29%) (100%)	35 (100%)	49 (100%)	7 (78%)	106 (78%)
Total	0(0%)	3(7%)	7(16%)	7(16%)	0(0%)	26(61%)	43	10(29%)	3(9%)	11(30%)	1(3%)	10(29%)	35	49	9	136
Group Total	43							35						49	9	136
Group %	31.62							25.74						36.03	6.62	

Max-maximum Min-Minimum

Supplementary Table 12: Distribution of false negatives from chromosome 7 in various categories

AmpliSeq																
	Library derived							Sequencing derived						Both	Unknown	Total
	Min coverage	Min coverage on either strand	Max strand bias	Min quality	Min relative read quality	Mixed	Total	Maximum homopolymer length	Excess outlier reads	Maximum common signal shift	Maximum reference / variant signal shift	Mixed	Total			
SNVs	0 (0%) (0%)	1(7%) (25%)	2(14%) (100%)	0 (0%) (0%)	0 (0%) (0%)	11(79%) (55%)	14 (50%)	0 (0%) (0%)	0 (0%) (0%)	3(100%) (23%)	0 (0%) (0%)	0 (0%) (0%)	3 (8%)	1 (4%)	0 (0%)	18 (19%)
Indels	0 (0%) (0%)	3(21%) (75%)	0 (0%) (0%)	2(14%) (100%)	0 (0%) (0%)	9(64%) (45%)	14 (50%)	16(43%) (100%)	4(11%) (100%)	10(27%) (77%)	1 (3%) (100%)	6(16%) (100%)	37 (93%)	27 (96%)	0 (0%)	78 (81%)
Total	0(0%)	4 (14%)	2(7%)	2(7%)	0(0%)	20 (71%)	28	16(40%)	4(10%)	13(33%)	1(3%)	6(15%)	40	28	0	96
Group Total	28							40						28	0	96
Group %	29.17							41.17						29.17	0.00	

SureSelect																
	Library derived							Sequencing derived						Both	Unknown	Total
	Min coverage	Min coverage on either strand	Max strand bias	Min quality	Min relative read quality	Mixed	Total	Maximum homopolymer length	Excess outlier reads	Maximum common signal shift	Maximum reference / variant signal shift	Mixed	Total			
SNVs	0 (0%) (0%)	0 (0%) (0%)	1(7%) (50%)	4(29%) (50%)	0 (0%) (0%)	9(64%) (69%)	14 (61%)	0 (0%) (0%)	0 (0%) (0%)	1 (100%) (13%)	0 (0%) (0%)	0 (0%) (0%)	1 (4%)	0 (0%)	0 (0%)	15 (39%)
Indels	0 (0%) (0%)	0 (0%) (0%)	1(11%) (50%)	4(44%) (50%)	0 (0%) (0%)	4(44%) (31%)	9 (39%)	13(54%) (100%)	1(4%) (100%)	7(29%) (88%)	1 (4%) (100%)	2(8%) (100%)	24 (96%)	32 (94%)	0 (0%)	65 (61%)
Total	0(0%)	0(0%)	2(9%)	8(35%)	0(0%)	13(57%)	23	13(52%)	1(4%)	8(32%)	1(4%)	2(8%)	25	32	0	80
Group Total	23							25						32	0	80
Group %	28.75							31.25						40.00		

Max-maximum Min-Minimum

Supplementary Table 13: Distribution of false negatives from chromosome 16 in various categories

**AmpliSeq**															
	Library derived							Sequencing derived						Both	Unknown	Total
	Min coverage	Min coverage on either strand	Max strand bias	Min quality	Min relative read quality	Mixed	Total	Maximum homopolymer length	Excess outlier reads	Maximum common signal shift	Maximum reference / variant signal shift	Mixed	Total			
SNVs	0 (0%) (0%)	0 (0%) (0%)	6(20%) (75%)	1(3%) (100%)	0 (0%) (0%)	23(77%) (68%)	30 (67%)	0 (0%) (0%)	0 (0%) (0%)	1(100%) (10%)	0 (0%) (0%)	0 (0%) (0%)	1 (5%)	1 (5%)	0 (0%)	32 (39%)
Indels	0 (0%) (0%)	2(13%) (100%)	2(13%) (25%)	0 (0%) (0%)	0 (0%) (0%)	11(73%) (32%)	15 (33%)	5(28%) (100%)	0 (0%) (0%)	9(50%) (90%)	0 (0%) (0%)	4(22%) (100%)	18 (95%)	18 (95%)	0 (0%)	51 (61%)
Total	0(0%)	2 (4%)	8 (18%)	1 (2%)	0(0%)	34 (76%)	45	5(26%)	0(0%)	10(53%)	0(3%)	4(21%)	19	19	0	83
Group Total	45							19						19	0	83
Group %	54.22							22.89						22.89	0.00	

**SureSelect**															
	Library derived							Sequencing derived						Both	Unknown	Total
	Min coverage	Min coverage on either strand	Max strand bias	Min quality	Min relative read quality	Mixed	Total	Maximum homopolymer length	Excess outlier reads	Maximum common signal shift	Maximum reference / variant signal shift	Mixed	Total			
SNVs	1 (100%) (0%)	0 (0%) (0%)	3(75%) (86%)	0 (0%) (0%)	0(0%) (0%)	13(76%) (76%)	17 (71%)	2 (50%) (22%)	1 (25%) (50%)	1 (25%) (10%)	0 (0%) (0%)	0 (0%) (0%)	4 (17%)	0 (0%)	0 (0%)	21 (31%)
Indels	0 (0%) (0%)	0 (0%) (0%)	1(25%) (14%)	0 (0%) (0%)	2 (29%) (100%)	4(57%) (24%)	7 (29%)	7(35%) (78%)	1(5%) (50%)	9(45%) (90%)	0 (0%) (0%)	3(15%) (100%)	20 (83%)	19 (100%)	0 (0%)	46 (69%)
Total	1(4%)	0(0%)	4(17%)	0 (0%)	2(8%)	17(71%)	24	9(38%)	2(8%)	10(42%)	0(0%)	3(13%)	24	19	0	67
Group Total	24							24						19	0	67
Group %	35.82							35.82						28.36		

Max-maximum Min-Minimum

Supplementary Table 14: Distribution of false negatives from chromosome 19 in various categories

**AmpliSeq**															
	Library derived							Sequencing derived						Both	Unknown	Total
	Min coverage	Min coverage on either strand	Max strand bias	Min quality	Min relative read quality	Mixed	Total	Maximum homopolymer length	Excess outlier reads	Maximum common signal shift	Maximum reference / variant signal shift	Mixed	Total			
SNVs	0 (0%) (0%)	1(2%) (11%)	6(15%) (75%)	3(7%) (75%)	0 (0%) (0%)	31(76%) (69%)	41 (62%)	0 (0%) (0%)	3 (60%) (50%)	2(40%) (32%)	0 (0%) (0%)	0 (0%) (0%)	5 (14%)	0 (0%)	0 (0%)	46 (37%)
Indels	0 (0%) (0%)	8(32%) (89%)	2(8%) (25%)	1(4%) (25%)	0 (0%) (0%)	14(56%) (31%)	25 (38%)	7(23%) (100%)	3(10%) (50%)	17(57%) (68%)	1 (3%) (0%)	2(7%) (91%)	30 (86%)	20 (100%)	2 (100%)	77 (63%)
Total	0(0%)	9 (14%)	8 (12%)	4 (6%)	0(0%)	45 (68%)	66	7(20%)	6(17%)	19(54%)	1(3%)	2(6%)	35	20	2	123
Group Total	66							35						20	2	123
Group %	53.66							28.46						16.26	1.63	
**SureSelect**																
	Library derived							Sequencing derived						Both	Unknown	Total
	Min coverage	Min coverage on either strand	Max strand bias	Min quality	Min relative read quality	Mixed	Total	Maximum homopolymer length	Excess outlier reads	Maximum common signal shift	Maximum reference / variant signal shift	Mixed	Total			
SNVs	0 (0%) (0%)	1 (2%) (0%)	4(10%) (86%)	2(5%) (14%)	0(0%) (0%)	34(83%) (81%)	41 (65%)	0 (0%) (0%)	0 (0%) (0%)	0 (0%) (0%)	0 (0%) (0%)	0 (0%) (0%)	0 (0%)	2 (6%)	0 (0%)	43 (39%)
Indels	0 (0%) (0%)	2(14%) (100)	1(7%) (14%)	4(29%) (86%)	0 (0%) (0%)	7(50%) (19%)	14 (35%)	10(42%) (100%)	3(13%) (100%)	10(42%) (100%)	0 (0%) (0%)	1(4%) (100%)	24 (100%)	29 (94%)	0 (0%)	67 (61%)
Total	0(0%)	3(5%)	5(9%)	6(11%)	0(0%)	41(75%)	55	10(100%)	3(100%)	10(100%)	0(0%)	1(100%)	24	31	0	110
Group Total	55							24						31	0	110
Group %	50.00							21.82						28.18	0	

Max-maximum Min-Minimum

Supplementary Table 15 : Distribution of false negatives from chromosome X in various categories

**AmpliSeq**																
	Library derived							Sequencing derived						Both	Unknown	Total
	Min coverage	Min coverage on either strand	Max strand bias	Min quality	Min relative read quality	Mixed	Total	Maximum homopolymer length	Excess outlier reads	Maximum common signal shift	Maximum reference / variant signal shift	Mixed	Total			
SNVs	0 (0%) (0%)	0 (0%) (0%)	0 (0%) (0%)	2(17%) (100%)	0 (0%) (0%)	10(83%) (50%)	12 (46%)	1(100%) (20%)	0 (0%) (0%)	0 (0%) (0%)	0 (0%) (0%)	0(0%) (0%)	1 (6%)	1 (7%)	0 (0%)	14 (25%)
Indels	1(8%) (100%)	3 (21%) (100%)	0 (0%) (0%)	0 (0%) (0%)	0 (0%) (0%)	10(71%) (50%)	14 (54%)	4(25%) (80%)	1(6%) (100%)	7(44%) (100%)	0 (0%) (0%)	4(25%) (100%)	16 (94%)	13 (93%)	0 (0%)	43 (75%)
Total	1(4%)	3(11%)	0(0%)	2(8%)	0(0%)	20(77%)	26	5(29%)	1(6%)	7(41%)	0(0%)	4(24%)	17	14	0	57
Group Total	26							17						14	0	57
Group %	45.61							29.82						24.56	0	
**SureSelect**																
	Library derived							Sequencing derived						Both	Unknown	Total
	Min coverage	Min coverage on either strand	Max strand bias	Min quality	Min relative read quality	Mixed	Total	Maximum homopolymer length	Excess outlier reads	Maximum common signal shift	Maximum reference / variant signal shift	Mixed	Total			
SNVs	0 (0%) (0%)	0 (0%) (0%)	0 (0%) (0%)	1(6%) (100%)	0 (0%) (0%)	15(94%) (79%)	16 (76%)	0 (0%) (0%)	0 (0%) (0%)	0 (0%) (0%)	0 (0%) (0%)	0 (0%) (0%)	0 (0%) (0%)	1 (5%)	0 (0%)	17 (34%)
Indels	0 (0%) (0%)	0 (0%) (0%)	1(20%) (100%)	0 (0%) (0%)	0 (0%) (0%)	4(80%) (21%)	5 (24%)	0 (0%) (0%)	1(13%) (100%)	3(37%) (100%)	0 (0%) (0%)	4(50%) (100%)	8 (100%)	19 (95%)	1 (100%)	33 (66%)
Total	0(0%)	0(0%)	1(5%)	1(5%)	0(0%)	19(90%)	21	0(0%)	1(13%)	3(37%)	0(0%)	4(50%)	8	20	1	50
Group Total	21							8						20	1	50
Group %	42							16						40	2	

Max-maximum Min-Minimu

Supplementary Table 16: False negative SNVs and indels in AmpliSeq and SureSelect

		Region	Library derived (%)	Sequencer derived (%)	Library + Sequencer (%)	Unknown (%)	Total (N)
**SNVs**	**AmpliSeq**						
	Chr1	TTR	74.5	18.2	1.8	5.5	55
		Specific	81.0	14.3	4.8	0.0	21
	ChrX	TTR	85.7	7.1	7.1	0.0	14
		Specific	75.0	25.0	0.0	0.0	4
	**SureSelect**						
	Chr1	TTR	93.3	0.0	0.0	6.7	30
		Specific	100.0	0.0	0.0	0.0	14
	ChrX	TTR	94.1	0.0	5.9	0.0	17
		Specific	92.9	0.0	7.1	0.0	14
**Indels**	**AmpliSeq**						
	Chr1	TTR	29.8	31.5	37.1	1.6	124
		Specific	30.9	33.8	30.9	4.4	68
	ChrX	TTR	32.6	37.2	30.2	0.0	43
		Specific	36.8	36.8	21.1	5.3	19
	**SureSelect**						
	Chr1	TTR	14.2	33.0	46.2	6.6	106
		Specific	13.0	43.5	41.3	2.2	46
	ChrX	TTR	15.2	24.2	57.6	3.0	33
		Specific	6.7	40.0	46.7	6.7	15

TTR - Total Target Region
Specific - Library specific region (AmpliSeq = 11.4 Mb, SureSelect = 14.1 Mb)
SNV- Single Nucleotide Variation

Supplementary Table 17: False negative SNVs and indels in AmpliSeq and SureSelect

	Chromosome	Library derived (%)	Sequencer derived (%)	Library + Sequencer (%)	Unknown (%)	Total (N)
SNVs	**AmpliSeq**					
	Chr1	74.5	18.2	1.8	5.5	55
	Chr7	77.8	16.7	5.6	0.0	18
	Chr16	93.8	3.1	3.1	0.0	32
	Chr19	89.1	10.9	0.0	0.0	46
	ChrX	85.7	7.1	7.1	0	14
	**SureSelect**					
	Chr1	93.3	0	0	6.7	30
	Chr7	93.3	6.7	0.0	0.0	15
	Chr16	81.0	19.0	0.0	0.0	21
	Chr19	95.3	0.0	4.7	0.0	43
	ChrX	92.9	0	7.1	0	14
Indels	**AmpliSeq**					
	Chr1	29.8	31.5	37.1	1.6	124
	Chr7	17.9	47.4	34.6	0.0	78
	Chr16	29.4	35.3	35.3	0.0	51
	Chr19	32.5	39.0	26.0	2.6	77
	ChrX	32.6	37.2	30.2	0	43
	**SureSelect**					
	Chr1	14.2	33	46.2	6.6	106
	Chr7	13.8	36.9	49.2	0.0	65
	Chr16	15.2	43.5	41.3	0.0	46
	Chr19	20.9	31.3	43.3	0.0	67
	ChrX	15.2	24.2	57.6	3	33

SNV- Single Nucleotide Variation

Supplementary Table 18: Distribution of false positives from chromosome 1

Categories	Strand bias	Read End	Low Quality	Homopolymer	Mixed alleles	Unknown	Total
**AmpliSeq**							
Strand bias	12						
Read End	3	2					
Low Quality	4	1	7				
Homopolymer	3	1	2	25			
Mixed alleles	3	0	0	6	12		
Unknown	0	0	0	0	0	14	
Total	25 (26.3%)	4 (4.2%)	9 (9.5%)	31 (32.6%)	12 (12.6%)	14 (14.7%)	95
**SureSelect**							
Strand bias	6						
Read End	0	0					
Low Quality	5	0	2				
Homopolymer	2	0	0	24			
Mixed alleles	0	0	0	2	11		
Unknown	0	0	0	0	0	22	
Total	13 (17.6%)	0 (0%)	2 (2.7%)	26 (35.1%)	11 (14.9%)	22 (29.7%)	74

*In AmpliSeq 1 FP was due to strand bias+homopolymer+read end, 1 FP =strand bias+read end+homopolymer and 1 FP =strand bias+read end+low quality. Total FPs – 98

Supplementary Table 19: Distribution of false positives from chromosome 7

Categories	Strand bias	Read End	Low Quality	Homopolymer	Mixed alleles	Unknown	Total
**AmpliSeq**							
Strand bias	6						
Read End	3	2					
Low Quality	1	0	2				
Homopolymer	3	0	0	15			
Mixed alleles	0	0	0	0	1		
Unknown	0	0	0	0	0	13	
Total	13(28.3%)	2 (4.3%)	2 (4.3%)	15 (32.6%)	1 (2.2%)	13 (28.3%)	46
**SureSelect**							
Strand bias	0						
Read End	0	0					
Low Quality	3	0	4				
Homopolymer	0	0	2	8			
Mixed alleles	0	0	0	1	4		
Unknown	0	0	0	0	0	15	
Total	3 (8.1%)	0 (0%)	6 (16.2%)	9 (24.3%)	4 (10.8%)	15 (40.5%)	37

*In AmpliSeq 1 FP = strand bias+low quality+homopolymer. Total FPs - 47

Supplementary Table 20: Distribution of false positives from chromosome 16

Categories	Strand bias	Read End	Low Quality	Homopolymer	Mixed alleles	Unknown	Total
**AmpliSeq**							
Strand bias	8						
Read End	1	3					
Low Quality	0	1	0				
Homopolymer	1	2	2	15			
Mixed alleles	0	0	0	0	1		
Unknown	0	0	0	0	0	9	
Total	10(23.3%)	6 (14%)	2(4.7%)	15 (34.9%)	1 (2.9%)	9 (20.9%)	43
**SureSelect**							
Strand bias	0						
Read End	0	0					
Low Quality	1	0	1				
Homopolymer	0	0	1	12			
Mixed alleles	0	0	0	1	1		
Unknown	0	0	0	0	0	12	
Total	1 (3.4%)	0 (0%)	2 (6.9%)	13(44.8%)	1 (3.4%)	12 (41.4%)	29

*In AmpliSeq 1 FP = read end+low quality+homopolymer. Total FPs - 44

Supplementary Table 21: Distribution of false positives from chromosome 19

Categories	Strand bias	Read End	Low Quality	Homopolymer	Mixed alleles	Unknown	Total
**AmpliSeq**							
Strand bias	34						
Read End	3	3					
Low Quality	5	2	4				
Homopolymer	9	2	1	21			
Mixed alleles	2	0	0	1	2		
Unknown	0	0	0	0	0	24	
Total	53 (46.9%)	7 (6.2%)	5(4.4%)	22 (19.5%)	2 (1.8%)	24 (21.2%)	113
**SureSelect**							
Strand bias	1						
Read End	0	0					
Low Quality	1	0	0				
Homopolymer	1	0	4	30			
Mixed alleles	0	0	0	0	5		
Unknown	0	0	0	0	0	31	
Total	3 (4.4%)	0 (0%)	4 (5.5%)	30(41.1%)	5 (6.8%)	31 (42.5%)	73

*In AmpliSeq 2 FP = strand bias +read end+homopolymer, 1 FP = strand bias+ homopolymer+mixed allele. Total FPs – 116

Supplementary Table 22: Distribution of false positives from chromosome X

Categories	Strand bias	Read End	Low Quality	Homopolymer	Mixed alleles	Unknown	Total
**AmpliSeq**							
Strand bias	**7**						
Read End	0	**1**					
Low Quality	3	0	**4**				
Homopolymer	2	0	0	**4**			
Mixed alleles	0	0	0	2	**2**		
Unknown	0	0	0	0	0	**19**	
Total	12 (27.3%)	1 (2.3%)	4 (9.1%)	6 (13.6%)	2 (4.5%)	19 (43.2%)	**44**
**SureSelect**							
Strand bias	**0**						
Read End	0	**0**					
Low Quality	0	0	**1**				
Homopolymer	0	0	0	**5**			
Mixed alleles	0	0	0	1	**3**		
Unknown	0	0	0	0	0	**20**	
Total	0 (%)	0 (%)	1 (3.3%)	6 (20%)	3 (10%)	20 (66.7%)	**30**

Supplementary Table 23: False positive SNVs and indels in AmpliSeq and SureSelect

		Region	Strand bias (%)	Read End (%)	Low Quality (%)	Homop-olymer (%)	Mixed alleles (%)	Unkn-own (%)	Total (N)
SNVs	**AmpliSeq**								
	Chr1	TTR	51.1	6.7	20.0	20.0	2.2	13.5	52
		Specific	48.1	11.1	14.8	22.2	3.7	15.6	32
	ChrX	TTR	61.1	5.6	22.2	11.1	0.0	43.8	32
		Specific	50.0	10.0	20.0	20.0	0.0	50.0	20
	**SureSelect**								
	Chr1	TTR	42.9	0.0	7.1	42.9	7.1	39.1	46
		Specific	18.0	0.0	9.1	54.5	18.2	47.6	21
	ChrX	TTR	0.0	0.0	50.0	50.0	0.0	90.0	20
		Specific	0.0	0.0	50.0	50.0	0.0	60.0	5
Indels	**AmpliSeq**								
	Chr1	TTR	5.6	2.8	0.0	61.1	30.6	16.3	43
		Specific	4.5	0.0	0.0	54.5	40.9	18.5	27
	ChrX	TTR	14.3	0.0	0.0	57.1	28.6	41.7	12
		Specific	0.0	0.0	0.0	66.7	33.3	50.0	6
	**SureSelect**								
	Chr1	TTR	4.2	0.0	0.0	58.3	37.5	14.3	28
		Specific	10.0	0.0	0.0	50.0	40.0	9.1	11
	ChrX	TTR	0.0	0.0	0.0	62.5	37.5	20.0	10
		Specific	0.0	0.0	0.0	50.0	50.0	33.3	3

TTR - Total Target Region
Specific - Library specific region (AmpliSeq = 11.4 Mb, SureSelect = 14.1 Mb)
SNV- Single Nucleotide Variation

Supplementary Table 24: False positive SNVs and indels in AmpliSeq and SureSelect

	Chrom-osome	Strand bias (%)	Read End (%)	Low Quality (%)	Homop-olymer (%)	Mixed alleles (%)	Unkn-own (%)	Total (N)
**SNVs**	**AmpliSeq**							
	Chr1	51.1	6.7	20	20	2.2	13.5	52
	Chr7	48.0	8.0	8.0	16.0	0.0	20.0	25
	Chr16	35.7	21.4	7.1	14.3	0.0	21.4	28
	Chr19	59.7	9.1	6.5	6.5	0.0	18.2	77
	ChrX	61.1	5.6	22.2	11.1	0	43.8	32
	**SureSelect**							
	Chr1	42.9	0	7.1	42.9	7.1	39.1	46
	Chr7	10.7	0.0	17.9	17.9	3.6	50.0	28
	Chr16	5.6	0.0	11.1	33.3	5.6	44.4	18
	Chr19	6.5	0.0	8.7	39.1	2.2	43.5	46
	ChrX	0	0	50	50	0	90	20
**Indels**	**AmpliSeq**							
	Chr1	5.6	2.8	0	61.1	30.6	16.3	43
	Chr7	5.0	0.0	0.0	50.0	5.0	40.0	20
	Chr16	19.4	0.0	0.0	47.2	5.6	27.8	36
	Chr19	0.0	0.0	0.0	78.6	7.1	14.3	14
	ChrX	14.3	0	0	57.1	28.6	41.7	12
	**SureSelect**							
	Chr1	4.2	0	0	58.3	37.5	14.3	28
	Chr7	0.0	0.0	11.1	44.4	33.3	11.1	9
	Chr16	0.0	0.0	0.0	63.6	0.0	36.4	11
	Chr19	0.0	0.0	0.0	44.4	14.8	40.7	27
	ChrX	0	0	0	62.5	37.5	20	10

SNV- Single Nucleotide Variation

Supplementary Table 25: Effect of increase in average read on per base coverage in AmpliSeq library

	270X	184X	92X	83X	44X
<5	400,116	605,213	1,102,034	722,020	3,188,163
5 - 10	220,562	459,515	2,543,570	869,884	6,629,198
10 - 400	45,660,202	53,520,615	53,993,427	56,217,098	49,058,469
>400	11,494,834	3,239,836	618,854	108,958	19,602
TPs	46,320	45,781	44,681	45,462	42,041
FNs	2,476	3,015	4,115	3,334	6,755
FPs	1,218	2,983	1,391	1,404	1,341
Sensitivity	94.93%	93.87%	91.57%	93.17%	86.16%
PPV	97.44%	93.88%	96.98%	97.00%	96.91%

TPs- True Positives
FNs- False Negatives
FPs- False Positives
PPV-Positive Predictive Value

Supplementary Table 26: Relative change in the number of TPs, FNs, and FPs with each step of optimization

**AmpliSeq**			
Optimization Steps	TPs	FNs	FPs
Regularization	1.6% (714)	-21.0% (-714)	2.1% (176)
High confidence region	-0.7% (-340)	-7.6% (-204)	-85.8 (-7 363)
Parameter settings	-0.3% (-143)	5.8% (143)	-27.4% (-334)
Total effect	0.5% (231)	-22.8% (-775)	-89.5% (-7 521)
**SureSelect**			
Optimization Steps	TPs	FNs	FPs
Regularization	1.4% (622)	-20.4% (-622)	2.5% (275)
High confidence region	-0.7% (-298)	-5.2% (-127)	-91.6% (-10 333)
Parameter settings	0% (-3)	0.1% (3)	-6% (-6)
Total effect	0.7% (321)	-24.4% (-746)	-91.4% (-10 064)

TPs- True Positives
FNs- False Negatives
FPs- False Positives
PPV-Positive Predictive Value
