# Supplementary material for: Evaluation of the Performance of AmpliSeq and SureSelect Exome Sequencing Libraries for Ion Proton
Source: Front Genet. 2019 Sep 25;10:856. doi: 10.3389/fgene.2019.00856 (PMC6774276; doi:10.3389/fgene.2019.00856)
Supplement: Supplementary file 2 [file DataSheet_2.pdf]

Supplementary datasheet:

Technical note on AmpliSeq Effective Target Regions provided by the manufacturer. The daSupplementary datasheet: Technical note on AmpliSeq Effective Target Regions provided by the manufacturer. The datasheet explains the strategies used to create AmpliSeq Effective Target Regions for AmpliSeq Exome.tasheet explains the strategies used to create AmpliSeq Effective Target Regions for AmpliSeq Exome.

ion torrent
life technologies

Technical Note

The Ion AmpliSeq™ Exome Hi-Q Effective Regions File

February 27, 2015

For Research Use Only. Not for use in diagnostic procedures.

About This Guide

Changes from previous version

Revision	Date	Description
A.0	February 27, 2015	Original

Contents

Background ... 4
Trimming Approach ... 4
Why trim and not remove amplicons? ... 5
Effect on medically relevant genes ... 5
Result of trimming (Mb of overlap), including a comparison ... 6
Results ... 6
Conclusion ... 7
Download Effective Regions BED file ... 7
View applied effective regions BED file in Torrent Suite™ Software ... 7

Background

The Ion AmpliSeq™ Exome Hi-Q Effective Regions file is a BED file in which poor performing flanking regions have been trimmed away from affected amplicons. To complement this effort, Torrent Variant Calling has also been optimized for the area covered by the Effective Regions File.

We extensively evaluated the Ion AmpliSeq™ Exome Hi-Q assay's performance and identified a set of regions where the Ion AmpliSeq™ Exome Hi-Q assay performs less well. These regions are typically at the edges of amplicons where sequence errors interact with alignment to cause false positives. Additionally, there are a few amplicons where the primers fail to successfully amplify and cause false negatives.

The Ion AmpliSeq™ Exome Hi-Q design targets exons and typically includes flanking regions since primer placement is often well into an intron. Most of the poorer performing regions are in intronic flanking regions, so we used a method of selectively excluding (trimming) such regions in Variant Calling.

Trimming Approach

The Torrent Suite Variant Caller (TVC) is highly optimized and determines whether a variant is truly present by using filters that filter out possible variants that are likely false. The metric used to measure performance is the regional density of filtered variants rather than the known truth. This metric guards against fitting to truth in a particular sample.

We applied the following rules:

1. Selectively trim 9.4 MB of intronic regions from the amplicons with the largest number of filtered candidates within introns (*i.e.,* we targeted false positives in introns),
2. Trim .4MB of non-Medical Exome Project exonic regions from amplicons with consistently very low read coverage (*i.e.*, we targeted false negatives),
3. Trim 1.4MB of non- Medical Exome Project exonic regions from amplicons with the largest number of filtered candidates within introns (*i.e.*, we targeted false positives in exons).

The following figure 1 illustrates these procedures.

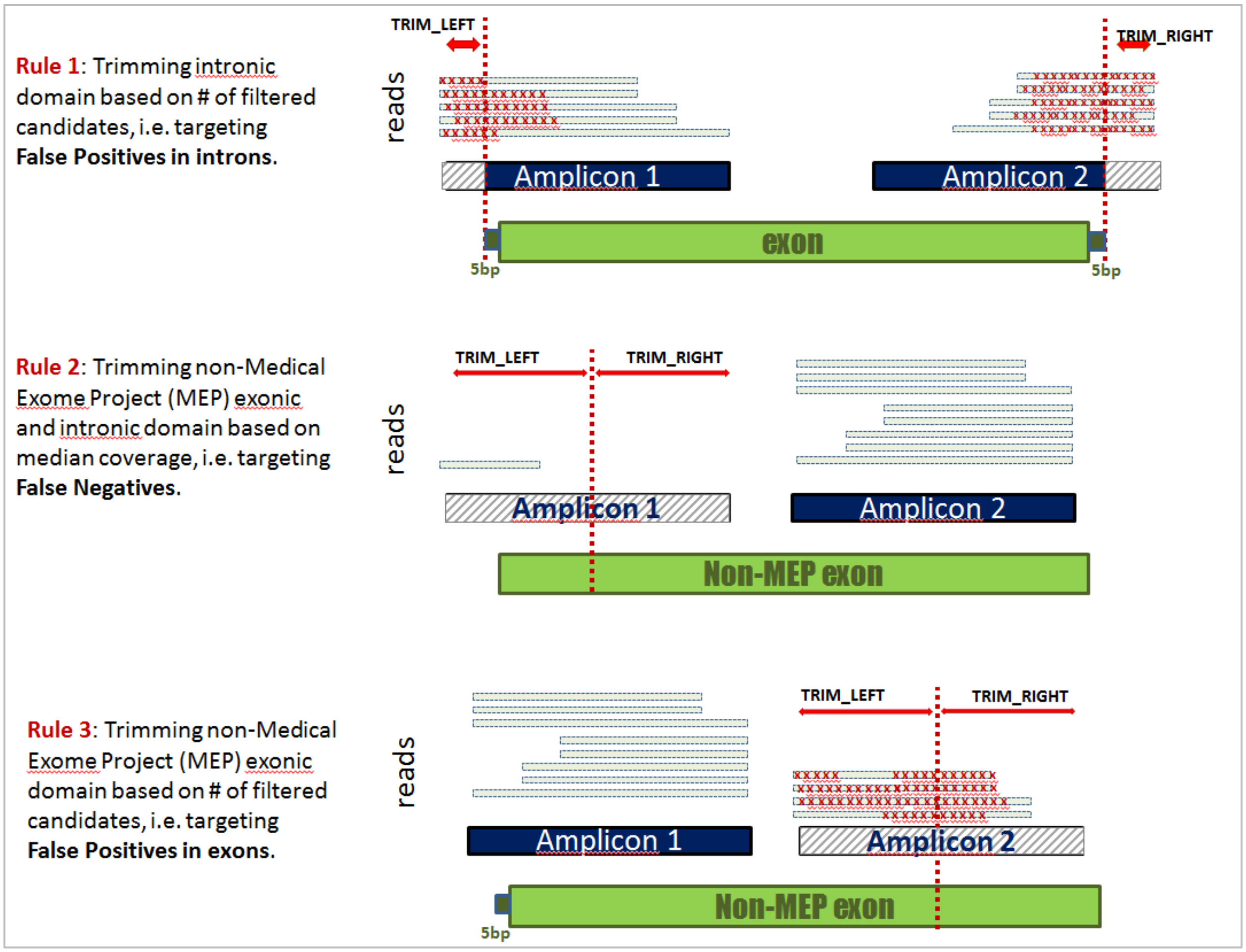

Figure 1 – Trimmed regions

Why trim and not remove amplicons?

We changed effective Ion AmpliSeq coverage by specifying TRIM_RIGHT/TRIM_END in the INFO field of the design regions file and modifying TVC to merge amplicons within (higher-quality) trimmed boundaries.

By modifying BED file, we maintained the integrity of existing Torrent Suite™ plugins as well as any internal Torrent Suite™ software that requires amplicon start/end positions — as these remain unchanged when a BED file including the TRIM directives is applied.

Effect on medically relevant genes

There are several lists of possibly medical relevant genes. We checked the result of trimming against a recent list that is the result of a curation effort. This list that includes 4,631 "genes associated with disease" presented during a talk given by Santani *et al.* the ACMG Annual Clinical Genetics Meeting, March 2014. This list was part of the Medical Exome Project, a 2013 initiative sponsored by Emory Genetics Laboratory, Children's Hospital of Pennsylvania and Harvard's laboratory of Molecular Medicine (a copy of the presentation slides is available at

http://ioncommunity.lifetechnologies.com/docs/DOC-9351). We also checked against claimed coverage for several other assays and the result is presented in the table below.

Result of trimming (Mb of overlap), including a comparison

	Estate	Exonic	Overlap with Medical Exome Project	Overlap with AmpliSeq Exome
**AmpliSeq baseline (prior to trim)**	57.9	36.7	10.0	57.9
**AmpliSeq post-trim**	46.5	34.8	10.0	46.5
**SureSelect**	50.6	34.0	9.2	40.7
**TrueSight**	11.9	11.3	8.8	11.3
**RapidCapture**	37.3	35.2	10.2	33.6

Note: there are 28 genes in the Medical Exome Project genes that are not present in Ion AmpliSeq Exome (trimmed or untrimmed), given in the table below:

C4A	H19	MIR4691	MSH5	RMST	UOX
C4B	MIAT	MIR486	NBEAP1	RNU4ATAC	WT1-AS
CEBPA-AS1	MIR17HG	MIR621	NCF1C	SNORD50A	XIST
DSCR8	MIR184	MIR96	OTOG	SULT1A3	
DUX4	MIR425	MIRLET7E	RMRP	TERC	

Note: No additional Medical Exome Project genes were affected by trimming.

Results

For a NA12878 sample using the NIST 2.18 "highly confident" truth set for this sample we see an improvement in sensitivity and specificity using Torrent Variant Caller v4.4 with the trimmed design bed file versus TVC v4.4 with the untrimmed design bed file. We also see a reduction in the number of variants called in the intersection of the NIST 2.18 truth set from ~37,000 to ~27,000. This lower number is primarily due to the removal of the poorly performing flanking intronic sequences and is accompanied by a marked reduction in Indel FP and SNP FP.

Using the NIST 2.18 truth set on an example NA12878 sample sequencing run in duplex with Hi-Q on Proton with and without trimming we see the following results:

TVC Version	SNP TP	SNP FP	SNP FN	SNP Sensitivity	SNP PPV	SNP FP/MB	InDel TP	InDel FP	InDel FN	InDel Sensitivity	InDel PPV	Indel FP/MB	Combined Sensitivity	Combined PPV	Combined FP/MB
4.4	26732	228	319	98.8	99.2	6.4	438	63	173	71.7	87.4	1.8	98.2	98.9	8.2
4.4 (no trim)	36086	483	627	98.3	98.7	11.1	748	135	504	59.7	84.7	3.1	97.0	98.3	14.1

Conclusion

We have excluded poorer performing regions of the Ion AmpliSeq™ Exome that are enriched for false positives or have coverage failures. We recommend you use our Effective Regions BED file to improve your sensitivity and specificity.

Download Effective Regions BED file

http://ioncommunity.lifetechnologies.com/docs/DOC-9350

Instructions for viewing the effective regions BED file in Torrent Suite™ Software

http://ioncommunity.lifetechnologies.com/docs/DOC-9327
